# Transcriptional regulation of the piRNA pathway by Ovo in animal ovarian germ cells

**DOI:** 10.1101/gad.352120.124

**Published:** 2025-02-01

**Authors:** Azad Alizada, Gregory J. Hannon, Benjamin Czech Nicholson

**Affiliations:** Cancer Research UK Cambridge Institute, Li Ka Shing Centre, University of Cambridge, Cambridge CB2 0RE, United Kingdom

**Keywords:** piRNAs, oogenesis, germ cells, transposon silencing, transcriptional regulation

## Abstract

The gene-regulatory mechanisms controlling the expression of the germline PIWI-interacting RNA (piRNA) pathway components within the gonads of metazoan species remain largely unexplored. In contrast to the male germline piRNA pathway, which in mice is known to be activated by the testis-specific transcription factor A-MYB, the nature of the ovary-specific gene-regulatory network driving the female germline piRNA pathway remains a mystery. Here, using *Drosophila* as a model, we combined multiple genomics approaches to reveal the transcription factor Ovo as regulator of the germline piRNA pathway in ovarian germ cells. Ectopic expression of Ovo in ovarian somatic cells activates germline piRNA pathway components, including the ping-pong factors Aubergine, Argonaute-3, and Vasa, leading to assembly of perinuclear cellular structures resembling nuage bodies of germ cells. We found that in ovarian somatic cells, transcription of *ovo* is repressed by *l(3)mbt*, thus preventing expression of germline piRNA pathway genes in the soma. Cross-species ChIP-seq and motif analyses demonstrate that Ovo is binding to genomic CCGTTA motifs within the promoters of germline piRNA pathway genes, suggesting a regulation by Ovo in ovaries analogous to that of A-MYB in testes. Our results also show consistent engagement of the Ovo transcription factor family at ovarian piRNA clusters across metazoan species, reflecting a deep evolutionary conservation of this regulatory paradigm from insects to humans.

## Abstract (precis)

In this study, Alizada et al. identify OVO as a key transcriptional regulator of germline piRNA pathway genes in the *Drosophila* ovary through direct binding to evolutionarily conserved promoter motifs (CNGTTA). Furthermore, OVO is regulated by L(3)mbt, establishing a mechanism for L(3)mbt-mediated repression of the germline piRNA pathway in ovarian somatic cells.

The PIWI-interacting RNA (piRNA) pathway is an evolutionarily conserved defense system in metazoans that silences transposons in gonads, thereby serving crucial functions in genomic stability, gametogenesis, and fertility (Czech et al. 2018; Ozata et al. 2019). Mutants affecting the piRNA pathway negatively impact the integrity of the germline genome, disrupt gamete development, and typically result in sterility (Carmell et al. 2007; Chen et al. 2007; Klattenhoff et al. 2007; Pane et al. 2007). This host–parasite conflict has driven the piRNA pathway to evolve several specialized adaptations to silence transposons in gonadal cells (Malone et al. 2009). The *Drosophila* ovary is one of the key models that has been used to decipher the workings of the piRNA pathway (Czech et al. 2013; Handler et al. 2013; Muerdter et al. 2013). Within the *Drosophila* ovary, unlike somatic follicular cells, the germline nurse cells use a germline-specific version of the pathway encompassing nuage bodies, the ping-pong cycle, and noncanonical transcription of dual-strand piRNA clusters (Czech et al. 2018; Ozata et al. 2019).

In the nuclei of *Drosophila* germ cells (Fig. 1A), transcription of piRNA precursors from dual-strand piRNA clusters is facilitated by Rhino (Rhi), a paralog of heterochromatin protein 1a that acts in complex with Deadlock (Del) and Cutoff (Cuff) (Klattenhoff et al. 2009; Mohn et al. 2014). Del interacts with Moonshiner (Moon), which recruits TBP-related factor 2 (Trf2) to enable dual-strand piRNA cluster transcription (Andersen et al. 2017). The resulting piRNA precursors are then exported out of the nucleus via a dedicated, noncanonical export machinery using nuclear export factor 3 (Nxf3), Bootlegger (Boot), UAP56, and Nxt1 (Zhang et al. 2012; Hur et al. 2016; ElMaghraby et al. 2019; Kneuss et al. 2019) and directed to germline-specific, perinuclear piRNA processing structures called nuage, where they are processed into functional piRNAs via the ping-pong cycle (Lim and Kai 2007).

**Figure 1.**
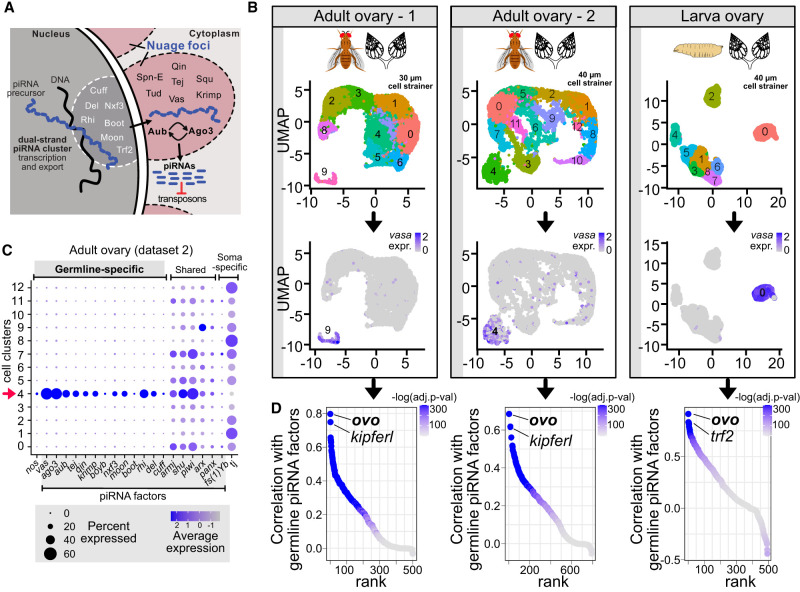
Ovo is the top transcription factor coexpressed with the germline piRNA pathway genes in the *Drosophila* ovary. (*A*) Model depicting the germline piRNA pathway in *Drosophila* germ cells. (*B*) UMAP clustering of *Drosophila* ovary single-cell RNA-seq data sets (adult ovary 1 from Rust et al. 2020; adult ovary 2 from Jevitt et al. 2020; LL3 larva ovary from Slaidina et al. 2020). Expression of the germline marker *vasa* is shown to mark germ cell clusters. (*C*) Dot plot showing expressions of the germline-specific, shared, and soma-specific piRNA pathway genes across the clusters identified in the adult ovary 2 data set (40 µm cell strainer; clusters from *A*; cluster 4 is the germline cluster). (*D*) Ranking of DNA-binding proteins and transcription factors by their average expression correlation values (Pearson's *r*) with the expression of the germline piRNA pathway factors *aub*, *vas*, and *ago3*. The color scale indicates correlation *P*-values adjusted with Bonferroni correction for multiple testing. Also see Supplemental Data Set S1.

In nuage bodies of *Drosophila* germ cells (Fig. 1A), ping-pong amplification operates via the PIWI proteins Argonaute-3 (Ago3) and Aubergine (Aub), which cleave piRNA precursor and transposon transcripts in an alternating loop, hence shaping and amplifying the piRNA pool against active transposons (Brennecke et al. 2007; Gunawardane et al. 2007). Ago3 in complex with a sense piRNA recognizes and cleaves cluster transcripts through sequence complementarity, generating antisense pre-piRNAs that are loaded into Aub. Antisense piRNA-loaded Aub in turn detects and cleaves target transposon mRNAs, thereby forming a new Ago3 complex loaded with a sense piRNA and completing the cycle (Brennecke et al. 2007; Gunawardane et al. 2007). This process is assisted by several nuage-localized protein components, including the DEAD-box RNA helicase Vasa (Vas), the putative nuclease Squash (Squ), and Tudor domain proteins Tejas (Tej), Tapas, Qin, Krimper (Krimp), and Spindle-E (Spn-E) (Lim and Kai 2007; Pane et al. 2007; Patil and Kai 2010; Zhang et al. 2011; Patil et al. 2014; Sato et al. 2015; Webster et al. 2015; Ryazansky et al. 2016). This is in sharp contrast to piRNA biogenesis in somatic cells, which lack nuage bodies and ping-pong amplification, and where piRNA precursors are transcribed from unistrand clusters (e.g., *flamenco*), exported to the cytoplasm by a canonical machinery, and processed into piRNAs via Zucchini-mediated biogenesis (Czech et al. 2018; Ozata et al. 2019).

The gene-regulatory network underlying the control of the germline-specific piRNA pathway in the ovaries of metazoan species have been largely unexplored. To date, clues from previous studies have pointed toward the existence of both positive and negative regulatory mechanisms. A study in mouse testes identified A-MYB as a transcriptional coordinator of piRNA factors such as *Piwil1* (*Miwi*) and germline pachytene piRNA clusters in male germ cells (Bolcun-Filas et al. 2011; Li et al. 2013), yet such regulatory pathways controlling the expression of the female germline piRNA pathway in the ovaries of metazoan species, including *Drosophila*, are not known. Nonetheless, earlier work in *Drosophila* ovaries has suggested negative regulation of germline piRNA factors in somatic cells (Janic et al. 2010; Sumiyoshi et al. 2016; Coux et al. 2018). Here, germline-specific components of the piRNA pathway such as *aub*, *ago3*, and *vas* were upregulated upon deletion of the tumor suppressor gene *lethal (3) malignant brain tumor* [*l(3)mbt*]; however, the mechanism underlying this regulation remains unclear. Several intriguing questions remain unanswered: What are the sex-specific differences in gene-regulatory networks underlying female and male germ cells? What are the mechanisms through which the coordinated transcription of germline piRNA factors and piRNA clusters are achieved? How does this vary between ovaries and testes, and what is their evolutionary conservation across the animal kingdom.

In this study, using *Drosophila* as a model, we uncovered key elements of the gene-regulatory network controlling the female germline piRNA pathway. In a systematic analysis integrating multiple approaches, including single-cell RNA-seq, ATAC-seq, and ChIP-seq, as well as depletion and overexpression screens, we identified the transcription factor Ovo as the key positive transcriptional regulator of the germline piRNA pathway in ovaries. Ectopic expression of Ovo in somatic cells activates expression of the germline piRNA pathway genes, including the ping-pong cycle components Aub, Ago3, and Vas, leading to formation of perinuclear cellular structures mimicking the nuage bodies of germ cells. This is orchestrated through binding of Ovo to conserved CCGTTA motifs within the promoters of these genes. We also reveal the mechanistic link between *l(3)mbt*, *ovo*, and the germline piRNA pathway genes. In addition, we show that Ovo-binding motifs are highly enriched within germline piRNA clusters in ovaries of metazoan species ranging from insects to humans. ChIP-seq experiments show that fly Ovo and its mouse and human orthologs, OVOL2, are recruited to the motifs within promoters of the piRNA pathway genes and ovarian piRNA clusters. Interestingly, the same CCGTTA motifs are also bound by the male-specific transcription factor A-MYB in testes of vertebrates to control the male germline piRNA pathway (Li et al. 2013). Our results indicate that Ovo is an ovary-specific counterpart of A-MYB, performing a role in ovaries analogous to that of A-MYB in vertebrate testes. Overall, our results reveal gene-regulatory interactions between the ovary-specific DNA-binding TFs and the CCGTTA *cis*-regulatory elements underlying the control of the germline piRNA pathway in ovaries across the metazoan species.

## Results

### Ovo is coexpressed with germline piRNA pathway genes in *Drosophila* ovary

Previous studies in mice identified the testis-specific transcription factor A-MYB as a regulator of male germline piRNA pathway genes (Bolcun-Filas et al. 2011; Li et al. 2013). To identify a potential master regulator of the female germline piRNA pathway in the *Drosophila* ovary, we performed a coexpression analysis using three separate single-cell RNA-seq data sets from *Drosophila* ovaries to determine the ovarian TFs coexpressed with the germline-specific piRNA pathway genes *aub*, *vas*, and *ago3* (Fig. 1B). Two of the scRNA-seq data sets were from adult ovaries (Jevitt et al. 2020; Rust et al. 2020), and one was from larval ovaries (Slaidina et al. 2020). Clustering of each of the data sets revealed distinct germline clusters showing specific expression of germline markers (e.g., *vas* and *nos*) and germline-specific piRNA pathway genes (i.e., *aub*, *vas*, *ago3*, *tej*, *qin*, *krimp*, *boyb*, *nxf3*, *moon*, *boot*, *rhi*, *del*, and *cuff*) (Fig. 1B,C; Supplemental Fig. S1A–D).

Correlation analysis (Pearson's *r*) identified *ovo* as the TF most significantly coexpressed with germline-specific piRNA pathway genes in all three data sets (Pearson's *r* > 0.7 in adult flies and Pearson's *r* > 0.9 in larva, adjusted *P* < 1.0 × 10^−300^) (Fig. 1D). The top coexpressed genes mostly represented germline-expressed and piRNA pathway-related genes (Supplemental Data Set S1). Interestingly, the second most significantly coexpressed TF in adult ovaries was *kipferl* (Fig. 1D), a recently identified zinc finger protein that interacts with Rhino and is required for piRNA production from most dual-strand piRNA clusters (Baumgartner et al. 2022). Of note, the second top-ranking TF in larval ovaries was *trf2* (Fig. 1D), which has been shown to assist Moon with transcription of dual-strand piRNA clusters (Andersen et al. 2017).

**Figure 2.**
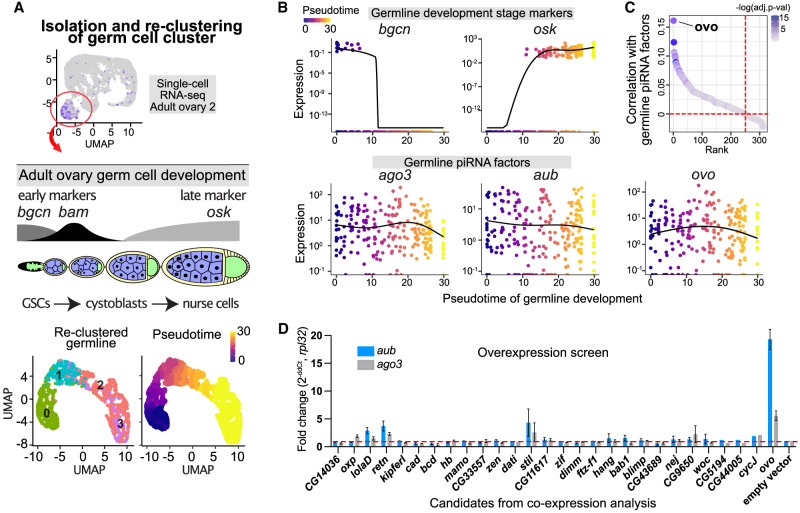
Overexpression screen of germline candidates coexpressed with piRNA pathway genes reveals Ovo as the positive regulator. (*A*) Diagram showing isolation and reclustering of the germ cells (cluster 4) from adult ovary single-cell RNA-seq (Supplemental Data Set S2) and computation of the pseudotime trajectory by rooting the *bgcn*-expressing germline stem cells (GSCs) as the starting point. (*B*) Expression pattern of the early (*bgcn*) and late (*osk*) stage markers of germline differentiation along the pseudotime trajectory of the germline development shown together with the germline piRNA pathway genes (*ago3* and *aub*) and the top coexpressed transcription factor *ovo*. (*C*) Ranking of the DNA-binding proteins and transcription factors by the average expression correlation (Pearson's *r*) with the germline piRNA pathway genes *aub*, *vas*, *qin*, and *ago3* within the reclustered germ cell cluster (cluster 4 in adult ovary scRNA-seq in Supplemental Data Set S2). Using the average of all germline piRNA pathway genes gave similar results. The color scale shows correlation *P*-values adjusted with Bonferroni correction for multiple testing. (*D*) Overexpression screen in ovarian somatic cells (OSCs) using the top coexpressed candidates (TFs and chromatin-binding proteins) from the coexpression analysis (RT-qPCR, 48–72 h after nucleofection in OSCs, *n* = 3 replicates from distinct samples; error bars indicate standard error of the mean). Overexpression of the Ovo-B isoform (matching NM_080338) is indicated for *ovo*. Also see Supplemental Data Set S1.

*Drosophila* ovaries are made up of ovarioles that comprise distinct stages of oogenesis, starting from the germline stem cells (GSCs) at the tip of germarium that produce cystoblasts, which give rise to nurse cells and the oocyte (Bastock and St Johnston 2008). Therefore, the germ cell cluster identified by scRNA-seq represents an aggregate of cells from distinct stages of germline development. To delineate the TFs coexpressed with germline piRNA pathway genes within germ cells across oogenesis stages, we isolated and reclustered the germ cell cluster and performed a correlation analysis specifically with the germ cells (Fig. 2; Supplemental Fig. S2).

Using early (*bgcn* and *bam*) and late (*osk*) markers of oogenesis (Ohlstein et al. 2000; Chen and McKearin 2003; Snee et al. 2007), we computed a pseudotime of germline development and tracked the expression pattern of the germline piRNA pathway genes from GSCs to nurse cells (Fig. 2A,B; Supplemental Fig. S2A,B). Germline piRNA pathway genes showed expression in both the early and middle stages of oogenesis captured by scRNA-seq data. We then searched for TFs showing the closest match in expression pattern. This germ cell cluster-specific correlation analysis revealed *ovo* as the top-ranking TF coexpressed with the germline piRNA pathway genes over the course of germline development (adjusted *P* < 3.4 × 10^−5^) (Fig. 2C; Supplemental Fig. S2C).

To functionally validate the top coexpressed gene-regulatory candidates, we performed an overexpression screen in ovarian somatic cells (OSCs) (Fig. 2D). Ectopic expression of *ovo* (isoform *ovo-B*, NM_080338 transcript) resulted in upregulation of the germline piRNA pathway genes in OSCs (*aub* ∼20-fold, *ago3* approximately fivefold, and *vas* approximately fivefold; *P* < 0.01, RT-qPCR), whereas other candidates had weaker or no effects on their expression (Fig. 2D).

### Ovo is the top germline-enriched transcription factor in the *Drosophila* ovary

Differential expression analyses of germline and somatic cell clusters in the three separate ovarian single-cell RNA-seq data sets showed *ovo* as the most enriched germline TF (Supplemental Fig. S3A). To validate this finding, we sought to experimentally identify all germline-enriched TFs within *Drosophila* ovaries that could potentially be responsible for promoting germline fate along with the germline piRNA program.

**Figure 3.**
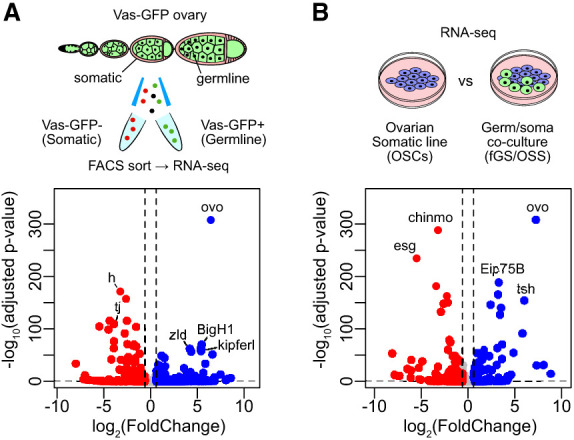
Ovo is the top enriched transcription factor in the *Drosophila* ovarian germ cells. (*A*) Differential gene expression of the DNA-binding TFs and chromatin proteins between the FACS-sorted *vas*-GFP^+^ (germline) and *vas*-GFP^−^ (somatic) cells from the transgenic *vas*-GFP fly ovaries (DESeq2; RNA-seq; *n* = 3 replicates from distinct samples). (*B*) Differential gene expression of the DNA-binding TFs and chromatin proteins between the ovarian somatic cells (OSCs) and the ovarian germ/soma coculture line (fGS/OSS) (DESeq2; RNA-seq; *n* = 4 replicates from distinct samples). Also see Supplemental Data Set S2.

To delineate germline-specific TFs, we first performed RNA-seq on FACS-sorted germline (*vas*-GFP^+^) and somatic (*vas*-GFP^−^) cells from *vas*-GFP *Drosophila* ovaries (Fig. 3A). A differential RNA-seq analysis between the sorted germline and somatic cells (DESeq2) revealed *ovo* as the top germline-enriched TF (∼87-fold, *P* < 1.0 × 10^−300^; *ovo-B* isoform matching NM_080338 transcript) (Fig. 3A). Cross-tissue RNA-seq analysis showed that ovaries are the most expressed site for *ovo* (Supplemental Fig. S3B). This result corroborates the hypothesis that *ovo* is the principal ovarian germline TF, potentially controlling a germline-specific expression program encompassing the piRNA pathway in ovarian germ cells. To confirm our findings, we performed RNA-seq on the ovarian germline/somatic coculture line (fGS/OSS) and compared it with purely somatic OSCs, which also revealed *ovo* as the most enriched germline-specific TF (∼155-fold, *P* < 1.0 × 10^−300^) (Fig. 3B).

The germline–somatic differential RNA-seq analysis of sorted cells from the *vas*-GFP *Drosophila* ovaries showed a total of 1019 germline-enriched and 1316 soma-enriched genes (log_2_FC > 1 and log_2_FC < −1, respectively; *P*adj < 0.01; DESeq2). Among the piRNA pathway genes, only one, *fs(1)Yb*, had a soma-specific expression, whereas 13 components of the germline piRNA pathway (namely, *aub*, *tej*, *ago3*, *vas*, *qin*, *boYb*, *krimp*, *boot*, *moon*, *nxf3*, *cuff*, *rhi*, and *del*) showed strong germline-specific expression (Supplemental Data Set S2) and thus could potentially be driven by Ovo.

### Ectopic *ovo* expression in OSCs activates germline piRNA pathway components

**Figure 4.**
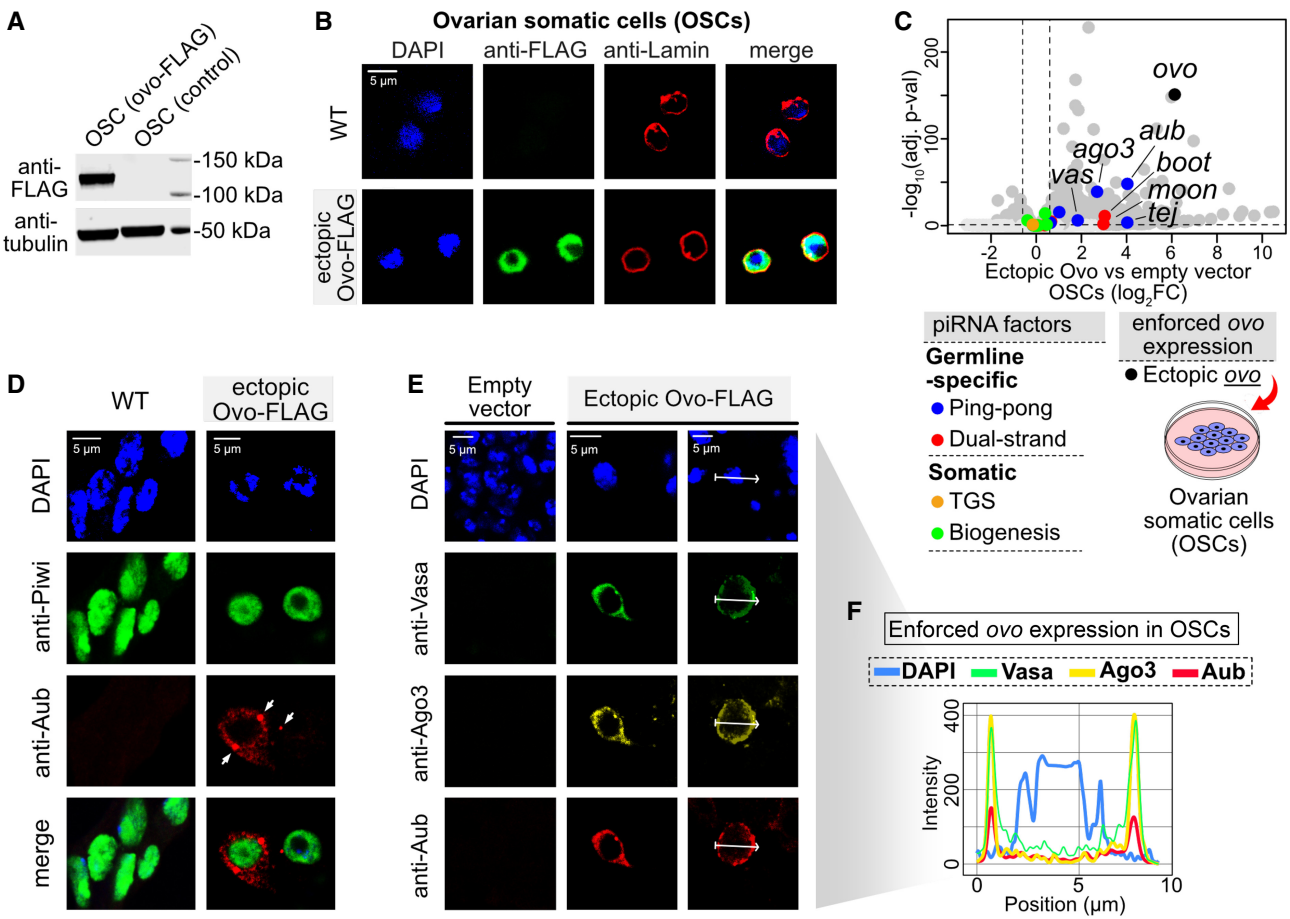
Ectopic expression of *ovo* in ovarian somatic cells (OSCs) activates the germline piRNA pathway components, leading to formation of cellular structures resembling nuage bodies of germ cells. (*A*) Western blot showing the presence of the Ovo-FLAG protein following ectopic expression in OSCs. (*B*) Immunofluorescence images showing the nuclear localization of the Ovo-FLAG protein in OSCs after nucleofection with the *ovo-FLAG* construct (48 h; Ovo-B isoform, NM_080338 transcript). (Blue) DAPI, (red) Lamin. (*C*) Differential gene expression between *ovo-FLAG* nucleofected OSCs relative to empty vector (DESeq2; RNA-seq *n* = 3 replicates from distinct samples; the piRNA pathway genes are labeled according to the color key). (TGS) Transcriptional gene silencing. (*D*) Immunofluorescence images showing the appearance of Aub, Ago3, and Vas as perinuclear nuage-like structures and foci (arrowheads) in the *ovo-FLAG* nucleofected OSCs. (Blue) DAPI, (green) Piwi, (red) Aub. (*E*) As in *D* but showing Vasa (green), Ago3 (yellow), and Aub (red). (*F*) Colocalization of Aub, Ago3, and Vas proteins within nuage-like bodies formed around the nuclei (DAPI) of the *ovo-FLAG* nucleofected OSCs. The fluorescence intensity along the white arrows in *E* is normalized to the highest value.

To determine the gene-regulatory effects of Ovo genome-wide, we performed RNA-seq in OSCs following ectopic *ovo* expression (overexpression vector carrying FLAG-tagged *ovo-B* isoform driven by the *act5C* promoter) and compared it with OSCs transfected with an empty vector. Western blotting confirmed the presence of the Ovo-B-FLAG protein at the expected size (∼114 kDa) (Fig. 4A). Immunofluorescence showed that overexpressed Ovo-B-FLAG localized to the nucleus (Fig. 4B; Supplemental Fig. S4A). Differential RNA-seq analysis (DESeq2) revealed that ectopic Ovo expression significantly upregulated ∼70% of the germline-specific piRNA pathway genes in OSCs; namely, *ago3*, *aub*, *tej*, *qin*, *vas*, *moon*, *boot*, and *nxf3* (>1.5-fold, adjusted *P* < 0.05) (Fig. 4C; Supplemental Fig. S4B,C; Supplemental Data Set S2). The strongest effects were observed for *aub* and *ago3* (16-fold and sixfold, adjusted *P* < 1.0 × 10^−41^) (Fig. 4C; Supplemental Fig. S4B,C). In contrast to components of the germline piRNA pathway, the soma-specific *fs(1)Yb* was downregulated upon Ovo overexpression, with no change in expression in all other somatic factors involved in biogenesis and transcriptional gene silencing (Fig. 4C; Supplemental Fig. S4C).

Using immunofluorescence, we observed the appearance of Aub, Ago3, and Vas proteins following ectopic Ovo expression in OSCs that assembled into perinuclear “nuage”-like structures (Fig. 4D–F; Supplemental Fig. S4D). Colocalization analysis showed that Aub, Ago3, and Vas proteins assembled as foci around nuclei in a ring-shaped manner (Fig. 4E,F), resembling the nuage structures of the *Drosophila* germ cells where piRNAs are processed by the ping-pong cycle. Of note, we confirmed that these “nuage”-like structures were distinct from the somatic Yb bodies of OSCs (Supplemental Fig. S4E).

A total of 693 genes was upregulated by ectopic Ovo expression in OSCs (log_2_FC > 0.6, *P*adj < 0.1; DESeq2), with 182 (∼26%) showing strong germline-enriched expression in ovaries (*vas*-GFP log_2_FC > 1, *P*adj < 0.01) (Supplemental Data Set S2). Ovo could account for the regulation of at least 18% of all strong germline-enriched genes in ovaries (182 out of 1019 genes). Ovo target genes in OSCs represented ∼70% (nine out of 13) of the germline-specific piRNA pathway genes and 16.8% of the germline-enriched genes. Notably, among the top germline Ovo targets was *nanos* (*nos*) (Supplemental Fig. S4F), a gene essential for germline formation and maintenance (Kobayashi et al. 1996; Forbes and Lehmann 1998). Therefore, our results suggest that Ovo controls germline piRNA pathway factors in addition to a more general role in ovarian germline development. Next, we sought to decipher mechanisms of Ovo regulation to understand how it activates piRNA factor expression exclusively in germ cells.

### L(3)mbt suppresses the germline piRNA pathway by repressing *ovo* in ovarian somatic cells

Early clues regarding the regulation of germline piRNA pathway components in *Drosophila* came from studies that deleted the *l(3)mbt* gene in somatic tissue, including the brain, ovary, and ovary-derived OSCs, which uniformly resulted in the upregulation of several germline-specific genes such as the ping-pong components Aub, Vas, and Ago3 (Janic et al. 2010; Sumiyoshi et al. 2016; Coux et al. 2018). The molecular mechanism underlying this activation has remained unknown. L(3)mbt has been shown to interact with chromatin and cause histone compaction, leading to suppression and insulation of gene expression (Trojer et al. 2007; Richter et al. 2011; Blanchard et al. 2014). Although a direct regulation by L(3)mbt could explain prior observations, we hypothesized that L(3)mbt deletion could also upregulate one or several specific TFs that in turn control the expression of germline piRNA pathway genes.

**Figure 5.**
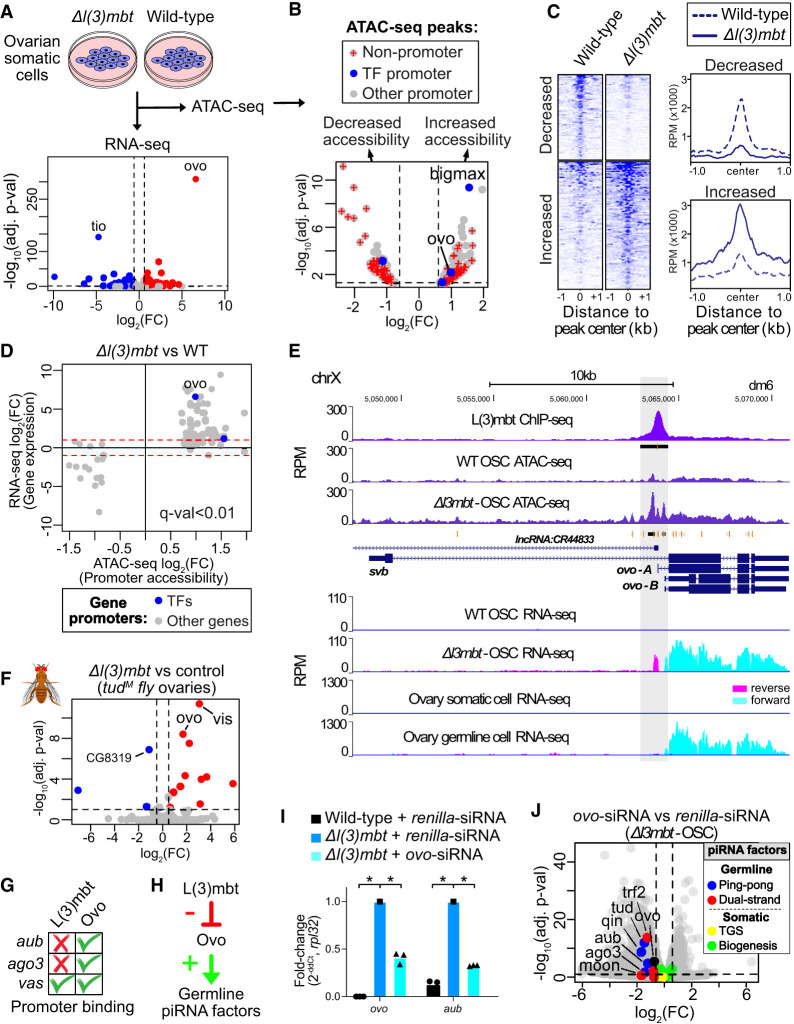
L(3)mbt negatively regulates the germline piRNA pathway via repression of *ovo* expression in somatic cells. (*A*) Differential gene expression of the DNA-binding TFs between the wild-type ovarian somatic cells (OSCs) and Δ*l(3)mbt* OSCs (DESeq2; RNA-seq; *n* = 4 replicates from distinct samples). (*B*) Differential chromatin accessibility between the wild-type OSCs and Δ*l(3)mbt* OSCs (DiffBind; ATAC-seq; *n* = 2 replicates from distinct samples). Gene promoters are defined as ATAC-seq peaks overlapping ±1 kb of the transcription start site (TSS). (*C*) Heat maps and genomic profiles of ATAC-seq reads corresponding to regions of increased and decreased chromatin accessibility (FDR *q* < 0.05; DiffBind). (*D*) All genes showing significant differential gene expression (RNA-seq; DESeq2; adjusted *P*-value < 0.01) and promoter chromatin accessibility (ATAC-seq; ±0.5 kb of gene TSS; DiffBind, FDR *q* < 0.01) between wild-type and Δ*l(3)mbt* OSCs. (*E*) Increased ATAC-seq peak accessibility (identified using DiffBind) at the *ovo* promoter region in Δ*l(3)mbt* OSCs (*n* = 2 replicates from distinct samples; merged) (our data) and L(3)mbt ChIP-seq from OSCs showing L(3)mbt binding at the region (*n* = 2 replicates from distinct samples; merged) (data from Yamamoto-Matsuda et al. 2022). RNA-seq tracks *below* show the expression of the germline *ovo* isoform in Δ*l(3)mbt* OSCs and *Drosophila* ovary germline cells (*n* = 4 replicates from distinct samples; merged) (our data). (*F*) Differential gene expression (DESeq2) between control and Δ*l(3)mbt* fly ovaries, both lacking germline due to *tud* maternal mutations (*tud*^*M*^; *n* = 3 replicates from distinct samples) (RNA-seq data from Coux et al. 2018). (*G*) Table summarizing L(3)mbt and Ovo ChIP-seq peaks within the promoters of the ping-pong genes *aub*, *ago3*, and *vas* (±1 kb of TSS). (*H*) Model for the L(3)mbt and Ovo regulation of the germline piRNA pathway genes. (*I*) Ovo siRNA knockdown experiments in Δ*l(3)mbt* OSCs (RT-qPCR; *n* = 3 replicates from distinct samples). (*) *P*-value: < 0.01, one-tailed two-sample *t*-test. (*J*) Volcano plot showing downregulation of the germline-specific piRNA pathway genes on day 2 of *ovo* siRNA knockdowns in Δ*l(3)mbt* OSCs using differential RNA-seq analysis (DESeq2) between *ovo* and *renilla* siRNA knockdowns (*n* = 3 replicates from distinct samples).

To determine the precise mechanistic link between L(3)mbt and germline piRNA pathway components, we performed RNA-seq (*n* = 3 replicates) and ATAC-seq (*n* = 2 replicates) on wild-type OSCs and OSCs carrying a deletion of *l(3)mbt*, referred to as Δ*l(3)mbt* (Fig. 5A; Sumiyoshi et al. 2016). We aimed to uncover regulatory elements, transcription factors, and genes that are differentially regulated and could be responsible for the activation of the germline piRNA pathway components upon L(3)mbt deletion.

Differential expression analysis of DNA-binding TFs (DESeq2 on RNA-seq data) between Δ*l(3)mbt* and wild-type OSCs revealed *ovo* as the top upregulated TF upon *l(3)mbt* deletion (∼97-fold, adjusted *P* value < 1.0 × 10^−300^) (Fig. 5A). The degree of *ovo* upregulation was markedly higher compared with other TFs (Fig. 5A). To assess whether this expression difference was due to increased accessibility at the *ovo* promoter upon *l(3)mbt* loss, we analyzed differential chromatin accessibility using ATAC-seq data (DiffBind) from Δ*l(3)mbt* and wild-type OSCs (Fig. 5B–D). This analysis uncovered 207 genomic regions with significantly increased accessibility upon *l(3)mbt* deletion (>1.5-fold, FDR *q* < 0.05), of which 111 were at gene promoters (±1 kb of TSS) (Fig. 5B,C). Out of these, 30 were germline-specific and included only one germline TF, *ovo* (approximately twofold, FDR *q* < 0.01) (Fig. 5D). The only other TF that showed increased promoter accessibility in Δ*l(3)mbt*-OSCs was *bigmax*, which was not germline-enriched in ovaries. Among piRNA pathway genes, only *vas* showed increased promoter accessibility in Δ*l(3)mbt*-OSCs and thus could potentially be a piRNA factor that is directly regulated by L(3)mbt. This is also supported by the L(3)mbt ChIP-seq binding signal at the *vas* promoter (±0.5 kb) (Fig. 5H) and disproportionately stronger upregulation of *vas* in *l(3)mbt* mutants compared with other piRNA factors or compared with ectopic Ovo alone in wild-type OSCs (Supplemental Data Set S2). Therefore, upregulation of *vas* in *l(3)mbt* knockouts and knockdowns is unlikely to be attributed to activated Ovo alone and is likely due to the lack of *l(3)mbt* repression over the *vas* promoter followed by activation by Ovo.

We speculated that direct binding of L(3)mbt to the *ovo* promoter could be responsible for the decreased chromatin accessibility in somatic cells, leading to its soma-specific transcriptional repression. Indeed, ChIP-seq data from OSCs showed strong L(3)mbt binding specifically at the promoter region of *ovo* (Fig. 5E), which increased in accessibility upon *l(3)mbt* deletion. This suggests that direct L(3)mbt binding to the *ovo* promoter in somatic cells represses its expression. Elimination of *l(3)mbt* consequently leads to increased accessibility at the *ovo* promoter and results in drastically increased expression of *ovo*, mimicking its germline expression in the *Drosophila* ovary (Fig. 5E). We further confirmed our results by reanalyzing recently published RNA-seq data from *l(3)mbt* knockdown in OSCs (Yamamoto-Matsuda et al. 2022), which also showed *ovo* as the most significantly upregulated gene (Supplemental Fig. S5A).

To extend our findings to an in vivo setting, we analyzed previously published RNA-seq data from Lehmann and colleagues (Coux et al. 2018) from *l(3)mbt* mutant and control ovaries of transgenic flies that lack germ cells due to maternal *tud* mutations (*tud*^*M*^). This allowed us to perform differential expression analysis between Δ*l(3)mbt* and control ovaries that were devoid of germline cells (Fig. 5F). This analysis identified *ovo* as the second most significantly upregulated TF upon *l(3)mbt* deletion in vivo within somatic cells of fly ovaries (Fig. 5F). It was previously shown that L(3)mbt forms a complex with Lint-O at chromatin to silence gene expression (Yamamoto-Matsuda et al. 2022). Differential expression analysis of RNA-seq data from *lint-O* and control knockdowns in OSCs revealed *ovo* as the top upregulated TF upon Lint-O depletion (Supplemental Fig. S5B), suggesting that Lint-O, together with L(3)mbt, forms a repressive complex at the *ovo* promoter in somatic cells, which we confirmed using Lint-O ChIP-seq data in OSCs (Supplemental Fig. S5C).

We then analyzed whether L(3)mbt could be exerting its effects on germline piRNA pathway genes, such as *aub*, indirectly via regulation of Ovo. Most importantly, using Ovo ChIP-seq, we observed strong Ovo binding at the promoters of germline-specific piRNA pathway genes where L(3)mbt binding was lacking (Fig. 5G; Supplemental Fig. S5F), suggesting that L(3)mbt was not directly controlling their expression. We therefore hypothesized that L(3)mbt indirectly represses the expression of germline-specific piRNA pathway genes in somatic cells via its regulation of *ovo* expression (Fig. 5H).

To establish a causal link, we performed siRNA knockdowns of *ovo* in Δ*l(3)mbt* OSCs, which express *ovo* due to the absence of L(3)mbt repression (Fig. 5I). Knockdown of *ovo* in Δ*l(3)mbt* OSCs resulted in the downregulation of germline piRNA pathway genes, including *aub* and *ago3* (*P* < 0.01, RT-qPCR), further supporting our hypothesis (Fig. 5I; Supplemental Fig. S5D). To validate our finding, we additionally performed RNA-seq on *ovo* siRNA knockdowns in Δ*l(3)mbt* OSCs (Fig. 5J; Supplemental Fig. S5E), which confirmed decreased expression of germline piRNA pathway genes, including *aub* (∼2.3-fold decrease, *P*adj = 2.8 × 1.0^−6^; DESeq2), *qin* (approximately threefold decrease, *P*adj = 2.6 × 1.0^−8^), and *ago3* (approximate3ly threefold decrease, *P*adj = 0.01) (Fig. 5J; Supplemental Fig. S5E).

Overall, in our analysis, we observed that ∼25% (*n* = 275) of all upregulated genes in Δl(3)mbt OSCs (*n* = 1085) were germline-enriched, representing ∼16% of all germline-enriched genes (*n* = 1700; log_2_FC > 0.6, *P*adj < 0.05). These genes could be either under a direct L(3)mbt repression [i.e., L(3)mbt promoter binding] or under an indirect repression via germline TFs such as Ovo that are repressed by L(3)mbt (Supplemental Data Set S2). Of the 315 L(3)mbt-repressed germline genes, 103 were upregulated by ectopic *ovo* expression alone in OSCs; therefore, ∼33% of the upregulated germline genes in Δ*l(3)mbt* OSCs were likely to be indirectly regulated through activation of *ovo* expression in the absence of *l(3)mbt* (Supplemental Data Set S2). RNA-seq of *ovo* siRNA knockdowns in Δ*l(3)mbt* OSCs revealed that ∼35% (*n* = 419 genes) of all L(3)mbt-repressed genes in OSCs (*n* = 1195) were downregulated by day 2 of *ovo* siRNA knockdown and thus could be accounted for by indirect regulation via Ovo (with ∼27%, 112 out of the 419 genes, being germline-enriched) (Supplemental Data Set S2). Out of a total of 2395 downregulated genes by day 2 of *ovo* siRNA treatment, 488 were germline-enriched, including germline piRNA pathway genes. Thus, we could conclude that at least 20% of all upregulated germline genes in Δ*l(3)mbt* OSCs were upregulated indirectly due to activation of Ovo. This fraction captured ∼62% of all germline-specific piRNA pathway components (e.g., *aub*, *ago3*, *tej*, *boot*, and *qin*). This proportion of upregulated germline genes remained at ∼20% (485 out of 2394) by day 4 of *ovo* siRNA treatment (second nucleofection on day 2) (Supplemental Data Set S2; Supplemental Fig. S5E).

### Ovo regulates the germline piRNA pathway genes via binding to conserved CNGTTA motifs

**Figure 6.**
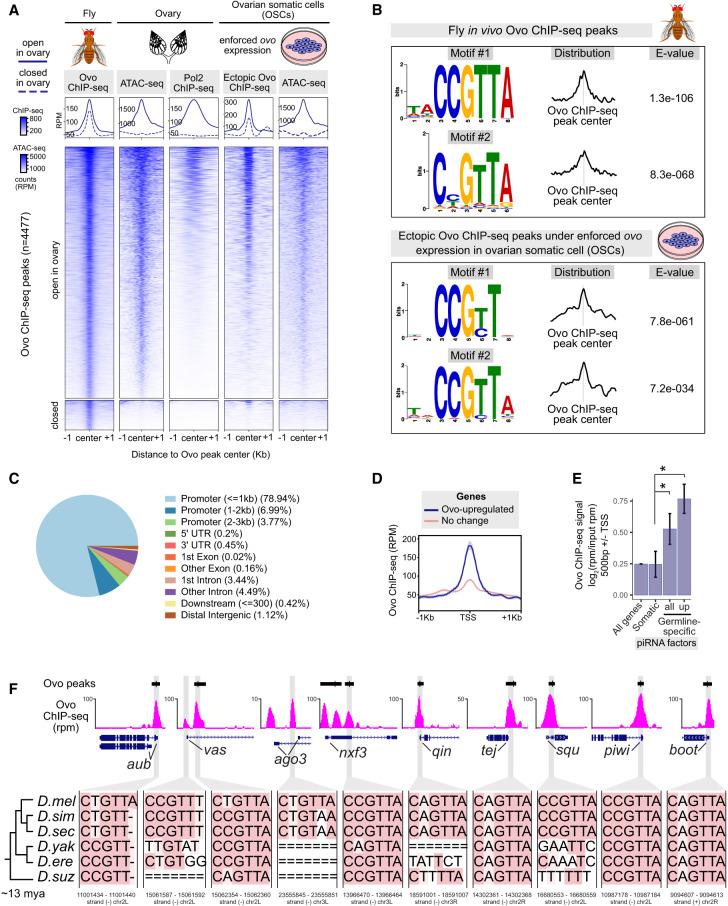
Ovo regulates germline piRNA pathway genes via binding to conserved CNGTTA motifs. (*A*) Heat map of fly Ovo ChIP-seq peaks (*n* = 2 replicates from distinct samples merged; ENCODE) corresponding to open and closed chromatin states in fly ovaries based on ovary ATAC-seq peaks (*n* = 2 replicates from distinct samples merged). The binding pattern of the ectopic Ovo ChIP-seq following ectopic *ovo* expression in OSCs (*n* = 2 replicates from distinct samples) and ATAC-seq accessibility of OSCs (*n* = 2 replicates from distinct samples) centered on the fly Ovo ChIP-seq peaks are shown by the heat maps at the *right*. The engagement of RNA polymerase II (Pol2) in ovaries is also depicted using the ovary Pol2 ChIP-seq signals (*n* = 1) (data from Andersen et al. 2017). (*B*) The top-scoring de novo motifs discovered within Ovo ChIP-seq peaks using MEME-ChIP. (*C*) Genomic annotations (UCSC; Ensembl genes; dm6) of the fly Ovo ChIP-seq peaks using ChIPseeker. (*D*) A genomic profile of fly Ovo ChIP-seq signals (RPM) within ±1 kb of the TSS of the upregulated, downregulated, and unresponsive genes following ectopic *ovo* expression in OSCs is shown. (*E*) Bar plots comparing fly Ovo ChIP-seq signals (input-normalized; RPM) between the promoters of the germline-specific and the somatic piRNA pathway genes. (*F*) The conservation of Ovo motifs within Ovo ChIP-seq peak summits at the germline piRNA pathway gene promoters is shown by multiple sequence alignments across six *Drosophila* species (27 way insect Multiz alignments and the phylogenetic tree from the UCSC genome browser conservation tracks; Ovo motif coordinates for the dm6 genome are shown *below*).

To capture Ovo binding events that lead to activation of the germline piRNA pathway genes, we performed Ovo ChIP-seq (*n* = 2) following ectopic *ovo* expression in OSCs (*n* = 4769 peaks, FDR < 0.05, more than fivefold enrichment). Using Ovo ChIP-seq data from adult female flies (ENCODE) (The modENCODE Consortium et al. 2010) we were also able to map Ovo binding events (*n* = 4477 peaks) across the *Drosophila* genome (Fig. 6A). We performed ATAC-seq in *Drosophila* ovaries to locate Ovo binding regions corresponding to open chromatin (*n* = 4007) and closed chromatin (*n* = 470) within ovaries (Fig. 6A). Ectopic Ovo expression in OSCs recapitulated ∼65% of the in vivo binding events observed in flies, of which the majority (∼93%) were within open chromatin in ovaries (Fig. 6A). Interestingly, Ovo binding sites at ∼72% of the germline piRNA pathway gene promoters in OSCs were restricted in chromatin accessibility before addition of Ovo (e.g., promoter regions of *aub*, *vas*, *qin*, *ago3*, *tej*, *nxf3*, and *boot*), whereas others, such as the promoter regions of *squ* and *piwi*, were already accessible. This could point to a pioneering factor-like ability of Ovo to bind motifs in closed chromatin configurations and potentially remodel and increase their accessibility, though this hypothesis requires further testing.

The top-scoring de novo motif within in vivo Ovo ChIP-seq peaks in flies was CCGTTA (MEME-ChIP, *e*-value = 1.3^−106^), while the secondary motif was CNGTTA (MEME-ChIP, *e*-value = 8.3^−68^) (Fig. 6B). As expected, the CCGTT core of the motif was the top hit within the ectopic Ovo ChIP-seq peaks following *ovo* overexpression in OSCs (MEME-ChIP, *e*-value = 7.8^−061^) (Fig. 6B), and CNGTT was the fourth strongest hit in vivo (Supplemental Fig. S6C). These results suggest that CCGTTA is the most preferred Ovo binding site in vivo, with changes to the second and last nucleotides often tolerated. The majority of Ovo binding events (∼79%) were proximal to promoters (<1 kb to the TSS), while only 1.12% were distal or intergenic (Fig. 6C). Approximately 95% of Ovo binding events at promoter-proximal regions corresponded to open chromatin in ovaries (based on ATAC-seq peaks) (Supplemental Fig. S6A,B). The genes upregulated in response to ectopic *ovo* expression in OSCs showed more than twofold stronger Ovo ChIP-seq binding signals and peak enrichments in their promoters (±1 kb of TSS, *P* < 0.01) compared with the genes that did not change in expression (Fig. 6D; Supplemental Fig. S6D). Correspondingly, higher numbers of Ovo motifs were observed at upregulated Ovo target gene promoters when compared with genes that did not change in expression (∼1.6-fold, *P* < 0.01) (Supplemental Fig. S6E,F).

Transcriptional activation by Ovo-B has been reported previously to occur via direct promoter binding at target genes (Bielinska et al. 2005; Benner et al. 2024). We therefore analyzed Ovo binding within promoters of the piRNA pathway genes (Fig. 6E,F). The average Ovo binding signal (input-normalized ChIP-seq) was more than twofold stronger (*P* < 0.05) at promoters of the germline-specific piRNA pathway genes when compared with somatic or shared piRNA factors (Fig. 6E). This was generally true for all germline genes, which showed on average approximately twofold stronger Ovo ChIP-seq signal (*P* < 4.1 × 1.0^−8^) at their promoters (±0.5 kb of TSS) when compared with the somatic or shared genes. We further validated these results by reanalyzing recently published Ovo-GFP ChIP-seq in ovaries (Benner et al. 2024), which also showed the strongest Ovo binding signals at germline piRNA pathway genes (Supplemental Fig. S6G).

Moreover, ∼90% of the germline-specific piRNA pathway genes and ∼30% of the germline-somatic shared piRNA pathway genes had an Ovo motif ±0.5 kb of their TSS overlapping an Ovo ChIP-seq peak summit (Supplemental Fig. S4C) compared with ∼22% of all germline-enriched genes. Multiple sequence alignments of Ovo binding sites within promoters of germline piRNA pathway genes showed high conservation of the CNGTTA motifs across six *Drosophila* species (*D. melanogaster*, *D. simulans*, *D. sechellia*, *D. yakuba*, *D. erecta*, and *D. suzuki*) sharing a common ancestor ∼13 million years ago (Suvorov et al. 2022), suggesting an evolutionarily conserved gene-regulatory mechanism behind Ovo interaction with CNGTTA motifs (Fig. 6F). These results indicate that direct binding to CNGTTA motifs is a major conserved mechanism through which Ovo controls expression of germline piRNA pathway genes in the *Drosophila* species.

### Vertebrate homologs of Ovo bind to ovarian piRNA pathway components

**Figure 7.**
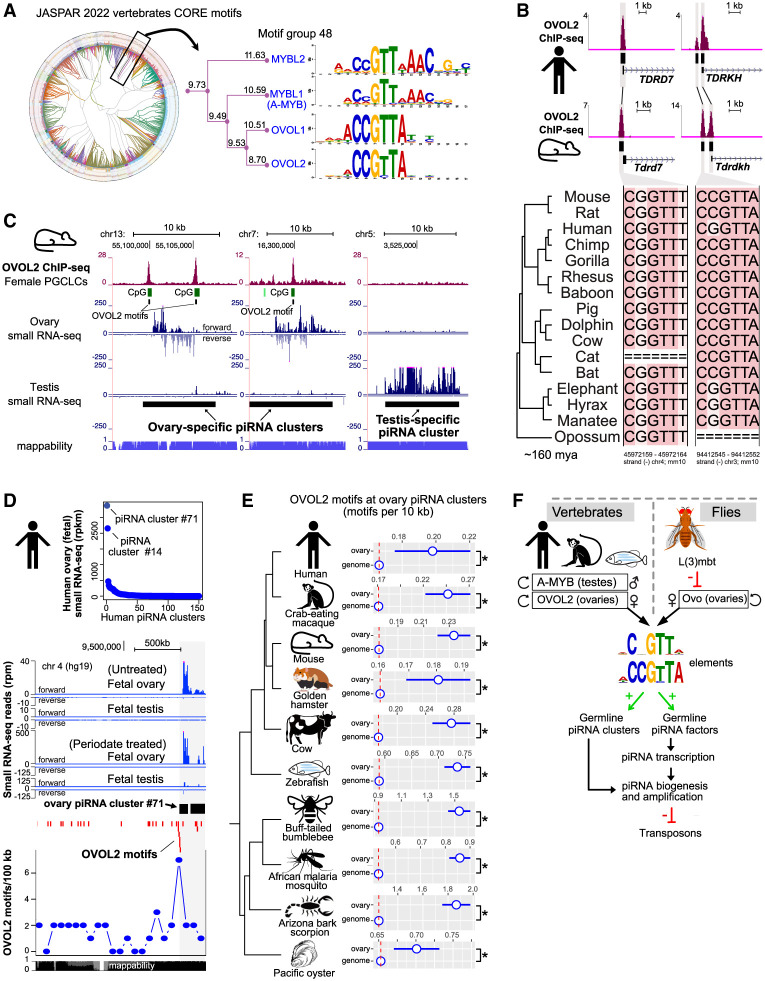
Ovo binding sites are hallmarks of ovarian piRNA pathway components across the genomes of metazoan species. (*A*) Radial tree clustering of all human TF motifs in the JASPAR database (JASPAR 2022 vertebrate CORE; RSAT matrix clustering) showing a zoomed-in view of motif group 48 containing the A-MYB and OVOL2 motifs. The information content of each branch is taken from the dynamic logo forest in JASPAR matrix clustering. (*B*) Human and mouse OVOL2 ChIP-seq showing OVOL2 binding events at orthologous genomic regions corresponding to promoters of the piRNA pathway genes *TDRD7* and *TDRKH* (RPM; merged *n* = 2 replicates from distinct samples). Mouse data are from day 2 of female mouse primordial germ cell-like cell (PGCLC) induction overexpressing mouse *Ovol2a* (Naitou et al. 2022), and human data are from an iPSC line overexpressing human OVOL2 (The ENCODE Project Consortium 2012). Multiple sequence alignments of OVOL2 motifs at peak summits indicate a high degree of conservation of Ovo binding sites across mammalian species (Multiz alignments of 46 vertebrates from UCSC conservation track; OVOL2 motif coordinates for mm10 are shown *below*). (*C*) OVOL2 ChIP-seq from female mouse PGCLCs (day 2 of PGCLC induction; overexpressing transgenic mouse *Ovol2a*) (data are from Naitou et al. 2022) showing OVOL2 binding to the CCGTTA motifs (OVOL2 motifs) within the mouse ovary-specific piRNA clusters (small RNA-seq data are from Aravin et al. 2008). (*D*) Ranking of the human piRNA clusters by their expression levels in human fetal ovaries (RPKM) (data are from Williams et al. 2015). The genomic browser *below* depicts OVOL2 motifs (red; UCSC squish track display) and the number of OVOL2 motifs per 100 kb at the top expressed human ovary piRNA cluster (#71). Human fetal ovary and fetal testis small RNA-seq signals show the relative production of the piRNAs from cluster #71 (RPM) (data are from Williams et al. 2015). Periodate treatment enriches for siRNAs and piRNAs. (*E*) Numbers of OVOL2 motifs per 10 kb compared with the genomic backgrounds across 10 metazoan species. Ovarian piRNA clusters were identified using ovary small RNA-seq data from each species (Supplemental Data Set S3). The classification tree is based on the NCBI taxonomy database. Error bars indicate standard error of the mean. (*) *P* < 0.05, Wilcoxon signed-rank test. (*F*) Model depicting a conserved regulation of the piRNA pathway via interactions of the CCGTTA motifs with Ovo family TFs in animal ovaries and A-MYB in animal testes.

Most vertebrates have three homologs of the fly *ovo* gene (e.g., mouse *Ovol1*, *Ovol2*, and *Ovol3*), with the *Ovol2* paralog involved in the development of primordial germ cells (PGCs) (Hayashi et al. 2017; Naitou et al. 2022). Clustering of all vertebrate TF motifs in the JASPAR database (2022 vertebrate CORE) revealed high similarities between the motifs of Ovo family TFs and, remarkably, A-MYB (Fig. 7A), a testis-specific TF previously reported to control transcription of both the germline piRNA pathway genes and piRNA clusters in male mice. We hypothesized that Ovo homologs could provide the ovary-specific equivalent of this regulation in females, controlling germline piRNA clusters in ovaries in addition to piRNA pathway genes via the same CCGTTA motifs.

To test this hypothesis, we used mouse OVOL2 ChIP-seq data from female mouse primordial germ cell-like cells (PGCLCs; day 2 of induction) under transgenic expression of mouse *Ovol2* (Naitou et al. 2022). We observed OVOL2 binding to CCGTTA motifs within or near promoters of the piRNA pathway genes (e.g., *Tdrkh*, *Tdrd7*, and *Pld6*) (Fig. 7B; Supplemental Fig. S7B), which also showed upregulation in response to *Ovol2* expression in mouse ESCs or PGCLCs (Supplemental Fig. S7B,C). To find out whether mouse OVOL2 also binds to ovary piRNA clusters, we defined ovary-specific, testis-specific, and shared piRNA clusters using small RNA-seq data from mouse ovaries and testes (more than one RPKM-normalized count mapping to the piRNA cluster regions [data from Aravin et al. 2008]; coordinates were obtained via proTRAC [Rosenkranz and Zischler 2012]) and checked OVOL2 binding at these regions (Fig. 7C; Supplemental Fig. S7A). Our analysis revealed strong OVOL2 binding events to CCGTTA motifs within regions corresponding to ovary-specific piRNA clusters (Fig. 7C; Supplemental Fig. S7A). These OVOL2 binding events within motifs were found near the ends or the center of ovary piRNA clusters (Fig. 7C; Supplemental Fig. S7A). OVOL2 ChIP-seq peaks showed a significant enrichment at ovarian piRNA clusters but were absent from testis-specific piRNA clusters (Fig. 7C; Supplemental Fig. S7A,D).

Next, we asked whether there was evidence of human OVOL2 engagement at human ovarian piRNA clusters. We exploited small RNA-seq data from human fetal ovaries (data from Williams et al. 2015) and analyzed OVOL2 motif occurrences within human ovarian piRNA clusters (Fig. 7D). Ranking of human ovary piRNA clusters with small RNA-seq expression using data and coordinates defined in Figure 7D and Supplemental Figure S7E and by Williams et al. (2015) revealed that the most highly expressed piRNA cluster in human fetal ovaries was cluster 71, which accounted for 54% of all piRNA reads in ovaries (Fig. 7D; Supplemental Fig. S7E). piRNA cluster 71 showed 2.5-fold enrichment for OVOL2 motifs over the genomic background (*P* < 0.01) (Fig. 7D). The enriched OVOL2 motifs were highly concentrated near the 5′ end of cluster 71, suggesting a potential impact on promoter activity (Fig. 7D). To determine whether OVOL2 was physically associated with these motifs, we used OVOL2 ChIP-seq data from human induced pluripotent stem cells (iPSCs; WTC11) (data from ENCODE; The ENCODE Project Consortium 2012). Indeed, OVOL2 ChIP-seq showed strong binding to the promoter region of ovarian piRNA cluster 71 (Supplemental Fig. S7F). Interestingly, piRNA cluster 14, the second highest ranking ovarian piRNA cluster, showed several strong OVOL2 binding regions across the cluster locus (Supplemental Fig. S7F).

Similar to the binding pattern observed for mouse OVOL2 ChIP-seq, human OVOL2 ChIP-seq showed binding near promoters of the piRNA pathway genes (e.g., *TDRKH*, *TDRD7*, *TDRD3*, and *PIWIL4*) when overexpressed in the human iPSCs (Fig. 7B; Supplemental Fig. S7B). Moreover, human OVOL2 binding near promoters of piRNA pathway genes (e.g., *TDRKH*, *PIWIL4*, and *TDRD7*) occurred at orthologous regions in the mouse genome where mouse OVOL2 showed corresponding binding events (Fig. 7B). These orthologous OVOL2 binding events occurred at Ovo motifs that were highly conserved across the mammalian species based on multiple sequence alignments (Fig. 7B). Overall, our results indicate that the Ovo TF family interactions with CCGTTA motifs in the regulation of ovarian piRNA pathway components is a gene-regulatory feature that is conserved from insects to vertebrates.

### Ovo motifs are hallmarks of ovarian piRNA clusters in metazoans

To test whether binding of Ovo to piRNA clusters in ovaries is a conserved feature of female germ cells, we analyzed Ovo motif enrichment within ovary piRNA clusters across multiple metazoan species. We characterized ovary piRNA clusters in species by their RPKM-normalized counts (more than one) mapping to the piRNA cluster regions (proTRAC piRNA cluster coordinates) (Rosenkranz and Zischler 2012; Rosenkranz et al. 2022) using ovary small RNA-seq data sets from humans (*Homo sapiens*), crab-eating macaques (*Macaca fascicularis*), mice (*Mus musculus*), golden hamsters (*Mesocricetus auratus*), cows (*Bos taurus*), zebrafish (Danio rerio), buff-tailed bumblebees (*Bombus terrestris*), African malaria mosquitoes (*Anopheles gambiae*), Arizona bark scorpions (*Centruroides sculpturatus*), and Pacific oysters (*Crassostrea gigas*). We then calculated the enrichments of OVOL2 motifs within the ovary piRNA clusters of each species. Our results revealed significant enrichment of OVOL2 motifs at ovary piRNA clusters in all 10 species when compared with genomic backgrounds (*P* < 0.05, Wilcoxon signed-rank test) (Fig. 7D).

Moreover, the Ovo motif enrichments at piRNA clusters were comparable with enrichments observed at promoter regions of the Ovo-upregulated target genes and germline-enriched genes and were significantly higher than the average signals observed at somatic-enriched genes or all genes in flies (Supplemental Fig. S6H). Overall, our results suggest a conserved regulatory mechanism using CCGTTA motifs underpinning the expression patterns of the germline piRNA pathway in metazoan species, where Ovo/OVOL2 interaction with the motifs in ovaries serves as a female counterpart of the male-specific regulation by A-MYB in testes (Fig. 7E).

## Discussion

Transcription factors and coactivators controlling the male germline piRNA pathway have been previously described in vertebrate testes (Bolcun-Filas et al. 2011; Li et al. 2013; Cecchini et al. 2023; Yu et al. 2023); however, a female-specific counterpart of such a regulatory network controlling the expression of the female germline piRNA pathway in insect and vertebrate ovaries has remained an enigma. The identification of ovary-specific TFs that control the germline-specific piRNA pathway in female germ cells provides crucial insights into the regulation of transposon repression during oogenesis in animals.

In this study, we uncovered Ovo as the principal regulator of ∼70% of the germline-specific piRNA pathway genes in *Drosophila*. The *ovo* locus encodes both somatic and germline isoforms that are driven by distinct promoters and were once thought to be two distinct genes: the somatic *shavenbaby* (*svb*) and the germline *ovo* (isoforms *ovo-A* and *ovo-B*) (Fig. 5E). The somatic *svb* is expressed only in embryonic, larval, and pupal epidermis cells, while the germline *ovo-B* is the major and the only essential isoform required for the viability of female *Drosophila* germ cells (Hayashi et al. 2017; Benner et al. 2024). Our results indicate that overexpression of *ovo-B* activates the germline piRNA pathway components in the ovarian somatic cells (Fig. 4), and *l(3)mbt* specifically represses the expression of *ovo-B* in the somatic cells by occupying the promoter region responsible for germline expression (Fig. 5E).

Our data point toward a gene-regulatory model of Ovo in female germ cells where transcription of germline piRNA pathway genes (e.g., *aub*, *ago3*, and *vas*) could be directed by Ovo family TFs, analogous to the model previously suggested for A-MYB in the regulation of the male germline piRNA pathway in mouse testes (Fig. 7F; Li et al. 2013). Similar to A-MYB, Ovo and its homologs autoregulate their own expression (Lü et al. 1998; Nair et al. 2007; Dragan et al. 2023) and, as our motif and ChIP-seq results reveal, show strong binding to germline piRNA pathway components. Our experiments show that Ovo is indeed able to activate expression of germline piRNA pathway components when ectopically expressed in fly somatic cells. This includes the nuage components Aub, Ago3, and Vas, which are activated in expression in somatic cells in the presence of ectopic Ovo and assemble to form cellular structures resembling the nuage bodies of germ cells (Fig. 4D–F). Of note, we found that upon ectopic Ovo expression, the germline-specific factors Rhino and Cuff are not upregulated in somatic cells, suggesting the involvement of additional regulatory mechanisms such as soma-expressed repressors or the lack of specific germline transcriptional coactivators in the somatic cells; however, these hypotheses require further experimental interrogation.

Our model is further supported by the multispecies analyses of OVOL2 ChIP-seq in mouse PGCLCs and human iPSCs revealing strong OVOL2 binding to the ovary piRNA clusters (Fig. 7), thus indicating a high degree of conservation of this gene-regulatory mechanism across the animal kingdom. Previous work analyzing ovarian piRNA clusters observed strong A-MYB motif enrichments at ovarian piRNA clusters in macaques (Roovers et al. 2015), leading the investigators to suggest a paradoxical involvement of the testis-specific A-MYB in driving transcription of ovarian clusters in ovaries. This enrichment is compatible with our findings given that OVOL2 and A-MYB use the same motifs, and these motifs would recruit OVOL2 in ovaries.

Ovo's conserved role in germline development in animals ranging from flies to mice is well known (Hayashi et al. 2017) despite germ cell development in flies and mammals following distinct pathways (Richardson and Lehmann 2010). In flies, the preformation model states that the germline is established through maternally deposited germ cell determinants within oocytes, whereas mammalian PGCs develop according to epigenetic/inductive mechanisms (epigenesis) where external cues dictate their developmental trajectory (Extavour and Akam 2003; Richardson and Lehmann 2010). Importantly, Ovo in flies is maternally deposited as a component of the germplasm and later pole cells, which establish the fly PGCs (Yatsu et al. 2008), whereas mammalian OVOL2 acts downstream from BMP signaling to control cell fate decisions during mammalian PGC specification in the epiblast (Zhang et al. 2013; Hayashi et al. 2017), thus suggesting that Ovo family TFs control animal PGC development and germline piRNA pathway expression via both intrinsic and inductive mechanisms.

Interestingly, ∼36% of the mouse OVOL2 binding sites were also occupied by A-MYB in testes, as both OVOL2 and A-MYB recognize the same core CCGTTA motif sequence (Fig. 7A). This could be a mechanism driving the expression of the shared germline piRNA clusters in both ovaries and testes (Supplemental Fig. S7A); however, other mechanisms such as motif affinity, chromatin accessibility, or methylation must account for expression of sex-specific piRNA clusters. In our motif analysis, we noticed a pseudopalindromic extension to the GTT core in the human A-MYB motif (Fig. 6A) that could prefer A-MYB over OVOL2 at testis-specific clusters. Additionally, we observed that ∼52% of the OVOL2 binding events within the mouse ovary piRNA clusters occurred at the CpG islands (Fig. 7C; Supplemental Fig. S7A), which could be linked to sex-specific DNA methylation patterns in developing PGCs. TF binding sites are enriched at hypomethylated regions that evade the first wave of default de novo DNA methylation (Molaro et al. 2011), which starts from day 13 of embryonic development (E13.5) in male mouse PGCs (Molaro et al. 2014) and coincides with the appearance of the prepachytene piRNAs (Aravin et al. 2008); however, female PGCs undergo de novo methylation only later after birth (Schaefer et al. 2007). Temporal differences in DNA methylation and binding patterns by sex-specific TFs that direct female PGC development such as OVOL2 could therefore protect against methylation to control sex-specific piRNA cluster transcription. Of note, the mouse *Ovol2* gene encodes both the repressor isoform *Ovol2a* and the activator isoform *Ovol2b*. The binding patterns between OVOL2A and OVOL2B ChIP-seqs were indistinguishable from each other at piRNA pathway components in mice; thus, the mechanism driving the ovary-specific piRNA pathway could also depend on the interplay of these isoforms.

In fly ovaries, Ovo is continuously present in the nucleus of germline cells, and maternal Ovo persists in the embryo until zygotic Ovo is expressed; thus, Ovo binding sites could be potentially marking genomic locations important during the transition from one generation to the next (Benner et al. 2024). Ovo persists in ovarian germ cells and plays a role in female germline sex determination by controlling expression of Otu and Sxl (Hempel et al. 2008). Our findings reveal its concurrent role in regulation of the female germline piRNA pathway. However, the testis-specific regulator controlling the male germline piRNA pathway in fly testes remains to be identified. The fly Myb TF is unlikely to act analogously to the vertebrate A-MYB, as it does not recognize the CCGTTA motifs (based on de novo motifs in ENCODE ChIP-seq data) and was reported to function as a weak repressor of piRNA factors in OSCs (Yamamoto-Matsuda et al. 2022); therefore, a different testis-specific TF candidate concurrently controlling spermatogenesis and the male piRNA pathway likely exists in fly testes. Interestingly, recent work identified the TF Trailblazer as an important regulator of the PIWI clade proteins Aub and Ago3 in male germ cells (Chen et al. 2024); however, it does not seem to control other piRNA genes or general germ cell factors.

Intriguingly, OVO and MYB family TFs belong to different lineages and harbor distinct DNA-binding domains yet are capable of binding and competing for the same genomic CCGTTA motifs (Nair et al. 2007), thus illustrating an example of convergent evolution where unrelated classes of DNA-binding domains evolve to bind to the same DNA elements. Under this model, the CCGTTA motifs represent *cis*-regulatory elements that interact with conserved female germline-specific Ovo family TFs in ovaries and male germline-specific A-MYB in testes of animals that, in combination with cofactors (such as TCFL5) (Cecchini et al. 2023; Yu et al. 2023) and other epigenetic mechanisms [e.g., chromatin accessibility, DNA methylation, histone marks, and L(3)mbt], control the germline piRNA pathway. Moreover, Ovo and Ovo-like TFs are comprised of both repressor and activator isoforms that could interplay to control the piRNA pathway in a sex- and tissue-specific manner. Overall, our results reveal a conserved gene-regulatory mechanism involving interactions of the ovary- and testis-specific TFs with the CCGTTA *cis*-regulatory elements behind the regulation of the germline piRNA pathway in animal ovaries and testes.

## Materials and methods

### Cell culture and treatments

Wild-type OSCs (*Drosophila* Genomics Resource Center [DGRC]288) (Saito et al. 2009), Δ*l(3)mbt*-OSCs (DGRC 289) (Sumiyoshi et al. 2016), and fGS/OSS (DGRC 191) (Niki et al. 2006) were purchased from the *Drosophila* Genomics Resource Center and cultured at 26°C in Shields and Sang M3 insect medium (Sigma-Aldrich S3652-6X1L) supplemented with 10% fetal bovine serum (Sigma-Aldrich F9665-500ML), 10% fly extract (DGRC 1645670), 0.6 mg/mL glutathione (Sigma-Aldrich G6013-25G), and 10 mU/mL human insulin (Sigma-Aldrich I9278-5ML). The fGS/OSS cells were cultured using 25% conditioned medium and passaged before reaching 50% confluency.

### Fly stocks and handling

All flies were kept at 25°C on standard cornmeal or propionic food. Control *w*^*1118*^ flies were available in the Hannon laboratory. *Vas*-GFP flies were reported by Bence et al. (2017) and obtained from the Bloomington *Drosophila* Stock Center (BDSC; 76126).

### Ovary dissections and cell dissociation

The ovary dissociation workflow was adapted from a previously published protocol (Slaidina et al. 2020). In brief, flies were fed with yeast extract 2–3 days before the dissections. Ovaries were dissected into ice-cold PBS, centrifuged at 500*g* for 5 min at 4°C, dissociated using 5 mg/mL trypsin (Sigma-Aldrich T1426-50MG) and 2.5 mg/mL collagenase-A (Sigma-Aldrich 10103586001) in Ringer's solution (600 µL for 148 ovaries) at 800 rpm for 1 h at 30°C in a thermomixer, passed through a 100 µm cell strainer followed by the addition of 400 µL of Schneider's medium (Thermo Fisher Scientific 21720001) containing 10% FBS, and centrifuged at 500*g* for 5 min at 4°C. The cell pellet was washed with 600 µL of PBS by centrifugation at 500*g* for 5 min at 4°C, and cells was resuspended in 500 µL of PBS. Dissociation of ∼30 ovaries resulted in ∼500,000 cells.

### FACS sorting

The *w*^*1118*^ control flies and transgenic *vas*-GFP flies were fed with yeast paste 3 days prior to dissections. Ovaries were dissected in ice-cold PBS, and cells dissociated as described above. Cells were resuspended in PBS and sorted into GFP-positive and GFP-negative populations using a FACS Aria cell sorter instrument (BD Biosciences). One-hundred-forty-eight ovaries from *vas*-GFP flies resulted in ∼36,000 *vas*-GFP-positive cells after FACS sorting, and 19 ovaries from the *w*^*1118*^ strain were dissociated and used as controls.

### RNA isolation and RT-qPCR

RNA was isolated using RNeasy the minikit (Qiagen 74106) with an RNase-free DNase set (Qiagen 79254) for DNase digestion during RNA purification as per the manufacturer's instructions. Reverse transcription was performed with 100 ng to 1 µg of RNA using SuperScript IV reverse transcriptase (Thermo Fisher Scientific 18090010) and oligo-dT primers. RT-qPCR was performed on 1:10 diluted cDNA using Fast SYBR Green master mix (Thermo Fisher Scientific 4385610) following the manufacturer's instructions on a Bio-Rad C1000 thermal cycler instrument. Primers were designed against exon–exon junctions for genes with introns, and *rpL32* was used as an internal standard (Supplemental Data Set S3). Relative expression was analyzed using the ΔΔ*Ct* method (Livak and Schmittgen 2001).

### RNA-seq

For total RNA-seq, ribosomal RNA depletion was performed using riboPOOLs for *Drosophila melanogaster* (Cambridge Bioscience) from 500 ng of total RNA input, as described previously (Munafò et al. 2021). For mRNA-seq, poly(A) selection was performed using the poly(A) mRNA magnetic isolation module (NEB E7490) with 500 ng of total RNA as input, as described previously (Fabry et al. 2019). Libraries were prepared using the NEBNext Ultra directional RNA library preparation kit for Illumina (NEB E7420L). Library quality was assessed using an Agilent 2100 bioanalyzer (high-sensitivity DNA kit). Libraries were quantified with a KAPA library quantification kit for Illumina (Kapa Biosystems KK4873) and sequenced on a NovaSeq6000 system with two 50 bp reads on an SP flow cell.

### ATAC-seq

For cell lines, 50,000 cells were used as described in the omni-ATAC-seq protocol (Corces et al. 2017). In brief, cells were lysed in ATAC resuspension buffer (RSB) containing 0.1% NP40, 0.1% Tween-20, and 0.01% digitonin and washed out with cold ATAC RSB containing 0.1% Tween-20. The transposition reaction was performed with 25 μL of 2× TD buffer and 2.5 μL of transposase (100 nM final) from the Illumina Tagment DNA enzyme and buffer small kit (Illumina 20034197), 16.5 μL of PBS, 0.5 μL of 1% digitonin, 0.5 μL of 10% Tween-20, and 5 μL of water and mixed at 1000 RPM in a thermomixer for 30 min at 37°C. For the whole-ovary ATAC-seq, the omni-ATAC-seq protocol was optimized by adjusting the numbers of ovaries used (six, 10, 20, and 30) and tagmentation time (30 min to 1 h). The Zymo DNA Clean and Concentrator-5 kit (Zymo D4014) was used for cleanup, and transposed fragments were amplified for five cycles [5 min at 72°C and 30 sec at 98°C (10 sec at 98°C, 30 sec at 63°C, and 1 min at 72°C for) × 5] using NEBNext 2× MasterMix (NEB M0541S) with Ad1_noMX and v2_Ad2.* indexing primers followed by qPCR amplification to determine the required additional cycle numbers. Amplified DNA was purified using the Zymo DNA Clean and Concentrator-5 kit (Zymo D4014) and eluted in 20 μL of water. AMPure XP beads (Beckman Coulter A63881) were used for double-sided bead purification. Fragments (100–600 bp) were selected using Blue Pippin (Sage Science NC1025035). The library fragment size distribution was assessed using an Agilent 2100 bioanalyzer on a high-sensitivity DNA kit. Libraries were quantified with a KAPA library quantification kit for Illumina (Kapa Biosystems KK4873) and sequenced on a NovaSeq6000 system with two 50 bp reads on an SP flow cell.

### ChIP-seq

For ectopic Ovo ChIP-seq, >30 × 10^6^ OSCs (*n* = 2) were nucleofected with an *ovo-B-FLAG* construct using 0.5–0.8 µg of plasmid per million cells, the nucleofector kit V (Lonza VVCA-1003), and the T-029 program on a Nucleofector 2b device (Lonza) and cross-linked 48 h later by covering the cells in 1% formaldehyde (FA) solution (50 mM HEPES-KOH, 100 mM NaCl, 1 mM EDTA, 0.5 mM EGTA, 1% FA) for 10 min at room temperature. Cell lysis was performed as described previously (Schmidt et al. 2009). Sonication was performed with 15–18 cycles of 10 sec on and 60 sec off (Fisherbrand Q705 Sonicator and 418-21 probe) in 3 mL of LB3 buffer (10 mM Tris–HCl at pH 8, 100 mM NaCl, 1 mM EDTA, 0.5 mM EGTA, 0.1% Na-deoxycholate, 0.5% N-lauroylsarcosine) in a 7 mL conical polypropylene tube to shear DNA into 100–500 bp fragments. ChIP was performed using 5–10 µg of primary mouse monoclonal anti-FLAG M2 antibody (Merck F1804-200UG) in 250 µL of LB3 with 1% Triton X-100 overnight at 4°C. Cross-links were reversed by incubation for 16 h at 65°C. Proteins and RNA were enzymatically digested. DNA purification was performed using phenol-chloroform extraction and ethanol precipitation. All of the purified DNA and 220 ng of whole-cell extract DNA were used to prepare ChIP-seq libraries using the NEBNext Ultra II DNA library preparation kit for Illumina (NEB E7645S) with PCR amplifications carried out for 16 cycles. The libraries were assessed for fragment size distribution using an Agilent 2100 bioanalyzer on a high-sensitivity DNA kit, quantified with the KAPA library quantification kit for Illumina (Kapa Biosystems KK4873), and sequenced on a NovaSeq6000 system with two 150 bp reads on an SP flow cell.

### Overexpression screen

Candidate genes were amplified from the *Drosophila melanogaster* ovary cDNA library using KOD Hot Start DNA polymerase (Merck Millipore 71086-4) and cloned into overexpression vectors under the *act5C* promoter with either N-terminal or C-terminal FLAG tags using Gibson assembly master mix (NEB E2611L) for 1 h at 50°C. Nine microliters of Mix & Go competent cells (strain DH5α) were thawed on ice and transformed with 1 μL of diluted (2.5× in water) Gibson assembly reaction by incubation for 5 min on ice before plating on LB plates containing the appropriate antibiotic. Colony PCR was performed to identify the colonies harboring the ligated constructs, followed by inoculation of the colonies into 3 mL of broth containing 100 μg/mL ampicillin and shaking overnight at 37°C. The constructs were purified using Qiagen Plasmid Plus kits and verified by Sanger sequencing. Constructs were then transfected into OSCs and Δ*l(3)mbt* OSCs with the nucleofector kit V (Lonza VVCA-1003) using the T-029 program and a Nucleofector 2b device (Lonza). Cells were passaged 1 day prior to transfections and were 70%–80% confluent at the time of transfections. The ectopic *ovo* overexpression vector carrying the FLAG-tagged *ovo-B* isoform matching NM_080338 transcript was used.

### siRNA knockdown experiments

Sense and antisense 21 nt siRNA sequences for target genes were designed using the DSIR tool (http://biodev.cea.fr/DSIR/DSIR.html; Supplemental Data Set S3). The designed sequences were ordered from IDT and resuspended in 400 μL of RNase-free water. Equal volumes of the resuspended sense and antisense siRNA were mixed and then added to an equal amount of 2× annealing buffer (60 mM potassium acetate, 200 mM HEPES at pH 7.5). The mix was boiled for 5 min at 75°C and then ramped down to 25°C (−0.1°C/sec) to anneal the siRNA sequences into the siRNA duplex (100 μM final concentration = 200 pmol). Two microliters of the final 100 μM siRNA duplex was mixed with 100 μL of nucleofection solution V (Lonza VVCA-1003) and transfected into 10 million cells using a Nucleofector 2b device and program T-029 (Lonza). Cells were plated into 12 well plates, and the media was changed after 24 h. Either RNA was harvested at 48 h or nucleofection was repeated after 48 h and RNA was harvested at 96 h.

### Western blots

Proteins were extracted from 1 × 10^6^ to 3 × 10^6^ cells, washed in PBS at 300*g* for 5 min, and lysed in ice-cold RIPA buffer (Thermo Scientific 89900) containing cOmplete protease inhibitor cocktail EDTA-free tablets (Roche; one tablet per 20 mL of RIPA) under rocking conditions for 30 min at 4°C. The lysate was centrifuged 20,000*g* for 20 min at 4°C, and supernatant was transferred to a new tube. The protein concentrations were measured using a Direct Detect spectrometer. Protein (15 mg/mL) was used with NuPAGE 4× LDS sample buffer and NuPAGE 10× sample-reducing agent (Thermo Fisher Scientific) in 20 μL of total volume and denatured for 10 min at 70°C followed by protein gel electrophoresis using 4%–12% NuPAGE and Bis-Tris in 1.5 mm 10 well miniprotein gels and XCell SureLock minicell electrophoresis system (Thermo Fisher Scientific) at 180 V for 1 h in a 4°C cold room. Precision Plus protein All Blue prestained protein standard (Bio-Rad 1610373) was run in parallel as a ladder. Blotting was performed for 7 min with iBlot 2 transfer stacks, nitrocellulose, and mini using an iBlot 2 gel transfer device. The nitrocellulose membrane was blocked for 1 h at room temperature under gentle agitation using TBS (LI-COR Biosciences). The membrane was rinsed with TBS buffer and incubated with primary mouse monoclonal anti-FLAG M2 antibody (1:2500; Merck F1804-200UG) and primary rabbit polyclonal anti-Tubulin (DM1A + DM1B; 1:5000; Abcam ab18251) in TBS with 0.1% Tween 20 (TBST) overnight at 4°C under gentle agitation. The membrane was washed three times for 5–10 min with TBST and incubated with the secondary antibodies IRDye 800CW donkey antimouse IgG secondary antibody (1:5000; LI-COR Biosciences 926-32212) and IRDye 680RD donkey antirabbit IgG secondary antibody (1:20,000; LI-COR Biosciences 926-68073) in TBST for 1 h room temperature under gentle agitation. The membrane was washed three times for 10 min with TBST and with TBS before image acquisition on an Odyssey CLx infrared imaging system (LI-COR Biosciences).

### Immunofluorescence (IF) and microscopy

OSC immunostainings were performed as described previously (Eastwood et al. 2021). Coverslips were coated with fibronectin (1:50 in PBS; Sigma-Aldrich F0895-1MG) for 1 h at 26°C in 6 well plates, and cells were plated on top. After 48 h, cells attached to coverslips were fixed in 4% PFA in PBS (500 µL per well) for 15 min at room temperature and rinsed three times with PBS. Cells were permeabilized with 0.2% Triton-X-100 in PBS for 10 min at room temperature and rinsed three times with PBS. Blocking was performed with 0.1% Tween-20 and 1% BSA in PBS. Primary mouse monoclonal anti-FLAG M2 (1:500; Merck F1804-200UG); primary rabbit monoclonal anti-FLAG (Cell Signaling Technology 14793S); primary rabbit polyclonal anti-Piwi, anti-Aub, or anti-Ago3 (1:500; available in-house; described by Brennecke et al. (2007); primary mouse monoclonal anti-Aub (1:500; Brennecke laboratory 8A3-D7)) (Senti et al. 2015); primary mouse monoclonal anti-Ago3 (1:500; Brennecke laboratory 7B4-C2); primary mouse monoclonal anti-Yb (Siomi laboratory, 8H12B12 and 180803); primary rat monoclonal anti-Vasa (Developmental Studies Hybridoma Bank [DSHB] AB_760351); and primary mouse monoclonal anti-Lamin Dm0 (DSHB adl67.10) antibodies were diluted in 0.1% Tween-20, 0.2% BSA, and 1× PBS (500 µL/well) and added onto coverslips in each well. Incubation with primary antibodies was performed overnight at 4°C under gentle agitation. Coverslips were washed three times for 5 min with PBST (1× PBS with 0.1% Tween-20) under gentle agitation. Goat antimouse IgG (H + L) highly cross-adsorbed secondary antibody AlexaFluor-647 (Thermo Fisher Scientific A-21236) and goat antirabbit IgG (H + L) cross-adsorbed secondary antibody AlexaFluor-488 (Thermo Fisher Scientific, A-11008) were diluted 1:500 in 0.1% Tween-20, 0.2% BSA, and 1× PBS and added onto coverslips (500 µL/well) for 1 h at room temperature under gentle agitation (covered with aluminum foil) followed by three 5 min washes with PBST. Coverslips were incubated with DAPI at 1:10,000 dilution in PBST for 10 min at room temperature (covered with aluminum foil) followed by two 5 min washes with PBST under gentle agitation. Coverslips were mounted on glass slides using a drop of ProLong Diamond antifade mountant (Thermo Fisher Scientific P36961). Imaging was performed on a Leica SP8 confocal laser scanning system, and image analysis was performed using Leica application suite X (v3.5.7.23225) and Huygens Professional (v20.04).

### ATAC-seq data analysis

The read quality was assessed with FastQC (v0.11.8). The paired-end reads were trimmed of adapter sequences TCGTCGGCAGCGTCAGATGTGTATAAGAGACAG and GTCTCGTGGGCTCGGAGATGTGTATAAGAGACAG using the Cutadapt tool (v1.18; default parameters). Burrows–Wheeler aligner (BWA; v0.7.17, bwa mem -M -t 4) (Li and Durbin 2009) was used to align the trimmed paired reads to the *D. melanogaster* genome (BDGP release 6 + ISO1 MT/dm6). Duplicates were marked using Picard tool (v2.9.0; MarkDuplicates, validation stringency = lenient). SAMtools (v1.9) was used for indexing and filtering (Li et al. 2009). The quality metrics for the aligned ATAC-seq reads were assessed using ataqv (v1.0.0) (https://github.com/ParkerLab/ataqv; Orchard et al. 2020). The ATAC-seq peaks were called with MACS2 (v2.1.1.20160309) using “‐‐nomodel ‐‐shift -37 ‐‐extsize 73” parameters and FDR cutoff of *q* ≤ 0.05 (Zhang et al. 2008). Differential accessibility analysis was performed using the DiffBind package (v3.0.15). RPKM-normalized bigWig files were generated using deepTools (v3.5.1; bamCoverage) (Ramírez et al. 2014). Peak intersections were performed using bedtools (v2.30.0) (Quinlan and Hall 2010). ATAC-seq heat maps and profiles were generated using the plotHeatmap and plotProfile tools in deepTools (v3.5.1). Genome browser visualizations were generated using the UCSC genome browser (Kent et al. 2002).

### RNA-seq data analysis

The reads were trimmed of adapter sequences using the Cutadapt tool (v1.18; -m 1 specified to not have reads trimmed to zero). The trimmed reads were aligned to the genome assemblies using the RNA-seq aligner STAR (v2.7.3a) (Dobin et al. 2013). Gene counts were calculated with the featureCounts tool (Subread package v1.5.3; -s 2 for stranded libraries prepared by the dUTP method) (Liao et al. 2014). *D. melanogaster* gene annotations were taken from Drosophila_melanogaster.BDGP6.28.100.gtf in the Ensembl genome database (Martin et al. 2023). SAMtools (v1.9) was used to merge bam files from replicates. Differential gene expression analysis was performed using DESeq2 (v1.30.1) (Love et al. 2014). The RPKM-normalized bigWig files were generated for each strand using the bamCoverage tool (‐‐filterRNAstrand specified for dUTP stranded libraries) in deepTools (v3.5.1). Raw data for publicly available RNA-seq reads were downloaded from the sources listed in Supplemental Data Set S3 and processed as above. Genome browser visualizations were generated using the UCSC genome browser.

### Single-cell RNA-seq analysis

The single-cell RNA-seq data matrices were downloaded from the sources listed in Supplemental Data Set S3 and clustered using the Seurat 4.0 toolkit (v4.3.0.1) (Hao et al. 2021). Normalizations were performed with the SCTransform() function. Differential expression analysis was performed using the FindMarkers() function. Pseudotime analysis was carried out with the Monocle 3 tool (v3.0) (Cao et al. 2019). Correlations were made with the Pearson correlation coefficients, and *P*-values were corrected for multiple testing using Bonferroni correction. The custom code for single-cell RNA-seq coexpression analysis is available at GitHub (https://github.com/alizadaa/Single-cell_RNA-seq_Co-expression_Analysis_Ovary).

### ChIP-seq data analysis

The reads were trimmed of adapter sequences using the Cutadapt tool (v1.18). The trimmed reads were aligned to the genome assemblies using Burrows–Wheeler aligner (BWA; v0.7.17, bwa aln). SAMtools (v1.9) was used for sorting, merging, and indexing. ChIP-seq peaks were called using the MACS3 (v3.0.0a6) callpeak command (-q 0.01), with data from ChIP input (the whole-cell lysate) used as the control (-c). The reproducibility of ChIP-seq peaks between replicates was evaluated using the irreproducible discovery rate (IDR) tool (v2.0.2) (Li et al. 2011). The RPKM-normalized bigWig files were generated using the bamCoverage tool in deepTools (v3.5.1) with the parameter “‐‐extendReads 120“ specified. The input-normalized bigWig files were generated using the bamCompare tool in deepTools (v3.5.1). Signals from bigWig files were quantified using the multiBigwigSummary tool in deepTools (v3.5.1). ChIP-seq heat maps and profiles were generated using the plotHeatmap and plotProfile tools in deepTools (v3.5.1). Raw data for publicly available ChIP-seq data sets were downloaded from the sources listed in Supplemental Data Set S3 and processed as described above. Genome browser visualizations were generated using the UCSC genome browser.

### Small RNA-seq and piRNA cluster analysis

The raw small RNA-seq reads from ovaries and testes of humans (*Homo sapiens*), crab-eating macaques (*Macaca fascicularis*), mice (*Mus musculus*), golden hamsters (*Mesocricetus auratus*), cows (*Bos taurus*), zebrafish (*Danio rerio*), buff-tailed bumblebees (*Bombus terrestris*), African malaria mosquitoes (*Anopheles gambiae*), Arizona bark scorpions (*Centruroides sculpturatus*), and Pacific oysters (*Crassostrea gigas*) were downloaded from the sources listed in Supplemental Data Set S3. Reads were trimmed of adapter sequences using Cutadapt tool (v1.18) and aligned to the respective genome assemblies using the RNA-seq aligner STAR (v2.7.3a). SAMtools (v1.9) was used to merge bam files from replicates. The RPKM-normalized bigWig files were generated using the bamCoverage tool in deepTools (v3.5.1). The piRNA cluster coordinates were taken from the piRNA cluster database (https://www.smallrnagroup.uni-mainz.de/piRNAclusterDB; Rosenkranz et al. 2022). Ovary and testis piRNA clusters were defined and ranked using the RPKM-normalized counts (more than one) mapping to the piRNA cluster regions as calculated from bigWig files of the ovary and testis small RNA-seq data sets using multiBigwigSummary tool in deepTools (v3.5.1). Genome browser visualizations were generated using the UCSC genome browser.

### Motif analysis, peak enrichments, and clustering

Motif scanning was performed using the FIMO tool in MEME suite (v5.4.1) (https://meme-suite.org/meme/index.html; Grant et al. 2011). De novo ChIP-seq motifs were generated using the MEME-ChIP tool in MEME suite (v5.4.1) (Ma et al. 2014). A list of *Drosophila* motifs was downloaded from the FlyFactorSurvey (http://pgfe.umassmed.edu/TFDBS) database (Zhu et al. 2011). fastaFromBed (v2.26.0) was used for conversion of bed file coordinates into fasta format using *D.mel* BDGP6.28.dna.toplevel genome (Bailey et al. 2015). Data were visualized with UCSC genome browser. ChIPseeker (v1.36.0) (Yu et al. 2015) was used to annotate the genomic features and distances based on the TxDb.Dmelanogaster.UCSC.dm6.ensGene annotation package. The radial tree clustering of human TF motifs was adapted from the JASPAR database (https://jaspar.elixir.no/matrix-clusters, JASPAR 2022 vertebrates CORE, RSAT matrix-clustering; Castro-Mondragon et al. 2017; Rauluseviciute et al. 2024. Orthologous regions between the species genomes were derived using the UCSC LiftOver tool (https://genome.ucsc.edu/cgi-bin/hgLiftOver; Hinrichs et al. 2006). The species classification tree was generated using the NCBI taxonomy database (Schoch et al. 2020) and visualized in TreeViewer (Bianchini and Sánchez-Baracaldo 2024). Multiple sequence alignments of motifs and phylogenetic trees were based on Multiz alignments (27 way insect alignment and 46 way vertebrates) (Blanchette et al. 2004) from the UCSC conservation tracks (Murphy et al. 2001; Kent et al. 2002; Hubisz et al. 2011).

### Data availability

RNA-seq, ATAC-seq, and ChIP-seq data generated in this study have been deposited to the GEO database under series GSE233246.

### Code availability

Custom code is available at GitHub (https://github.com/alizadaa/Single-cell_RNA-seq_Co-expression_Analysis_Ovary).

## Supplemental Material

Supplement 1

Supplement 2

Supplement 3

Supplement 4

Supplement 5

Supplement 6

Supplement 7

Supplement 8

Supplement 9

Supplement 10
