# Supplementary material for: Transcriptional regulation of the piRNA pathway by Ovo in animal ovarian germ cells
Source: Genes Dev. 2025 Feb 1;39(3-4):221–41. doi: 10.1101/gad.352120.124 (PMC11789646; doi:10.1101/gad.352120.124)
Supplement: Supplement 4 [file Supplemental_Figure_S1.pdf]

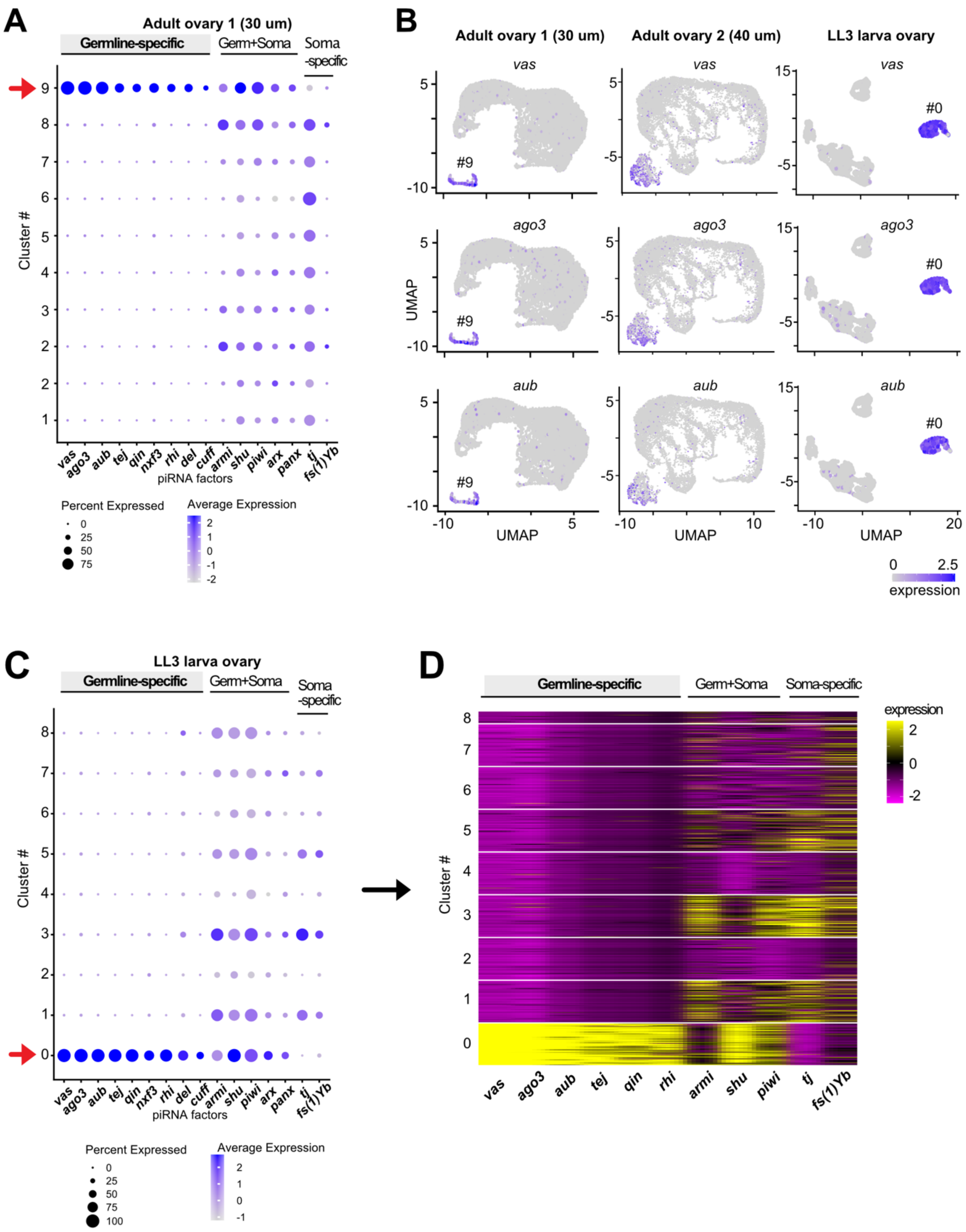

Supplemental Figure S1. ***Drosophila*** **ovary single-cell RNA-seq datasets reveal specific expression of the germline piRNA pathway genes in the ovarian germ cell clusters.**
(A-B) Dot plot and feature plots showing the expression of the germline-specific, shared, and soma-specific piRNA pathway genes across the cell clusters identified from the adult ovary dataset 1 (30 µm cell strainer; cluster 9 is the germline cluster; data from (Rust et al. 2020)) and (B-D) across the clusters identified from the late third-stage larva (LL3) ovary (40 µm cell strainer; cluster 0 is the germline cluster; data from (Slaidina et al. 2020). Red arrows indicate the germ cell clusters).
