# Supplementary material for: Transcriptional regulation of the piRNA pathway by Ovo in animal ovarian germ cells
Source: Genes Dev. 2025 Feb 1;39(3-4):221–41. doi: 10.1101/gad.352120.124 (PMC11789646; doi:10.1101/gad.352120.124)
Supplement: Supplement 5 [file Supplemental_Figure_S2.pdf]

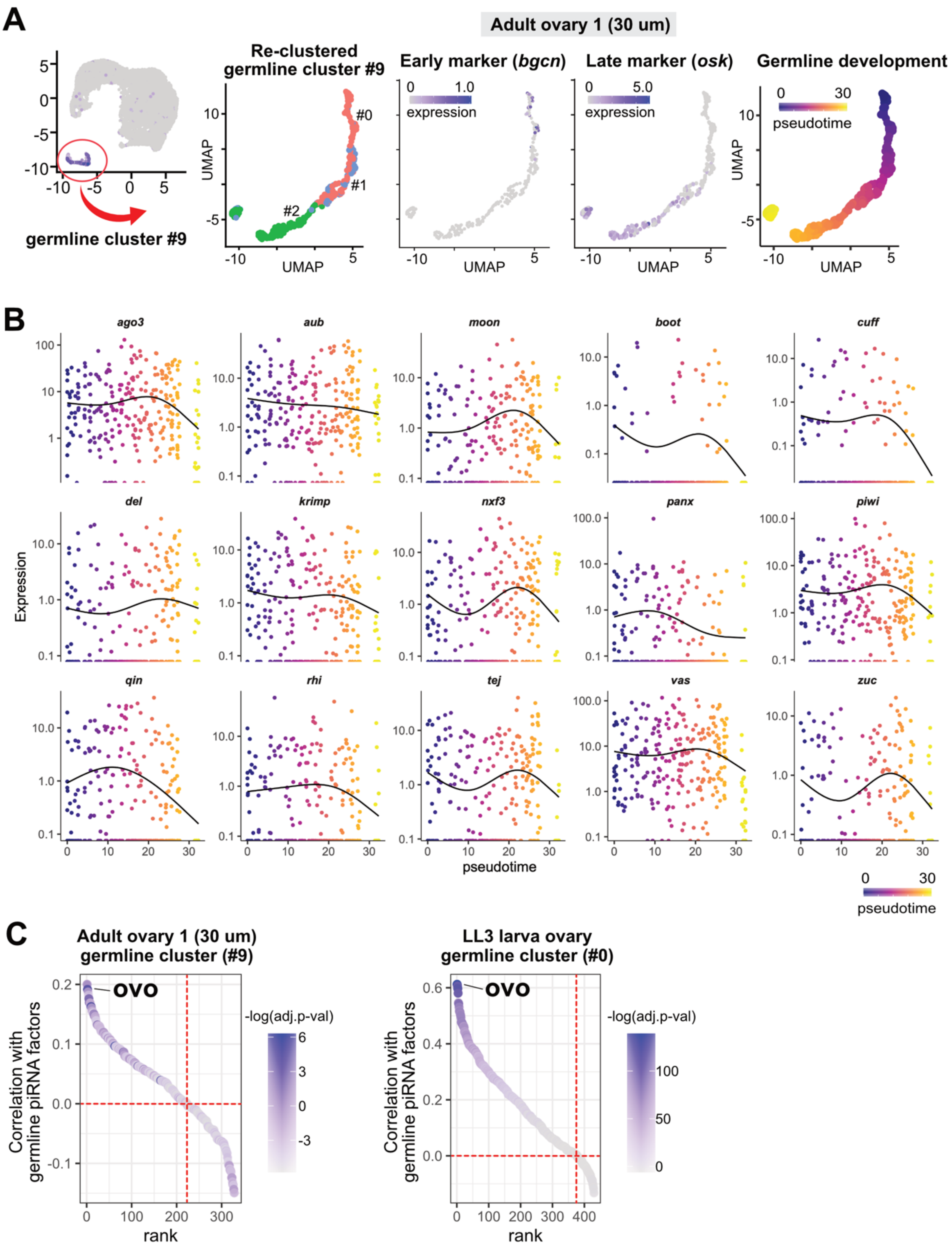

Supplemental Figure S2. Ovo is the top transcription factor co-expressed with germline piRNA pathway genes throughout germ cell stages (also see Supplemental Dataset 1).
(A) UMAP re-clustering of the germline cluster 9 from the adult ovary 1 dataset and pseudotime trajectory of germline development computed by rooting the early *bgcn*-expressing germline stem cells (GSCs) as the starting point. (B) The expression pattern of the piRNA pathway genes along the pseudotime trajectory of the germline development in the adult ovary 1 dataset. (C) Ranking of the DNA-binding transcription factors (TFs) by the average expression correlation (Pearson's r) with the germline piRNA pathway genes *aub*, *vas*, *qin*, and *ago3* in the re-clustered germline cluster 9 of the adult ovary 1 (left) and the re-clustered germline cluster 0 of the larva ovary (right). The colour scales show correlation p-values adjusted with Bonferroni correction for multiple testing.
