# Supplementary material for: Transcriptional regulation of the piRNA pathway by Ovo in animal ovarian germ cells
Source: Genes Dev. 2025 Feb 1;39(3-4):221–41. doi: 10.1101/gad.352120.124 (PMC11789646; doi:10.1101/gad.352120.124)
Supplement: Supplement 6 [file Supplemental_Figure_S3.pdf]

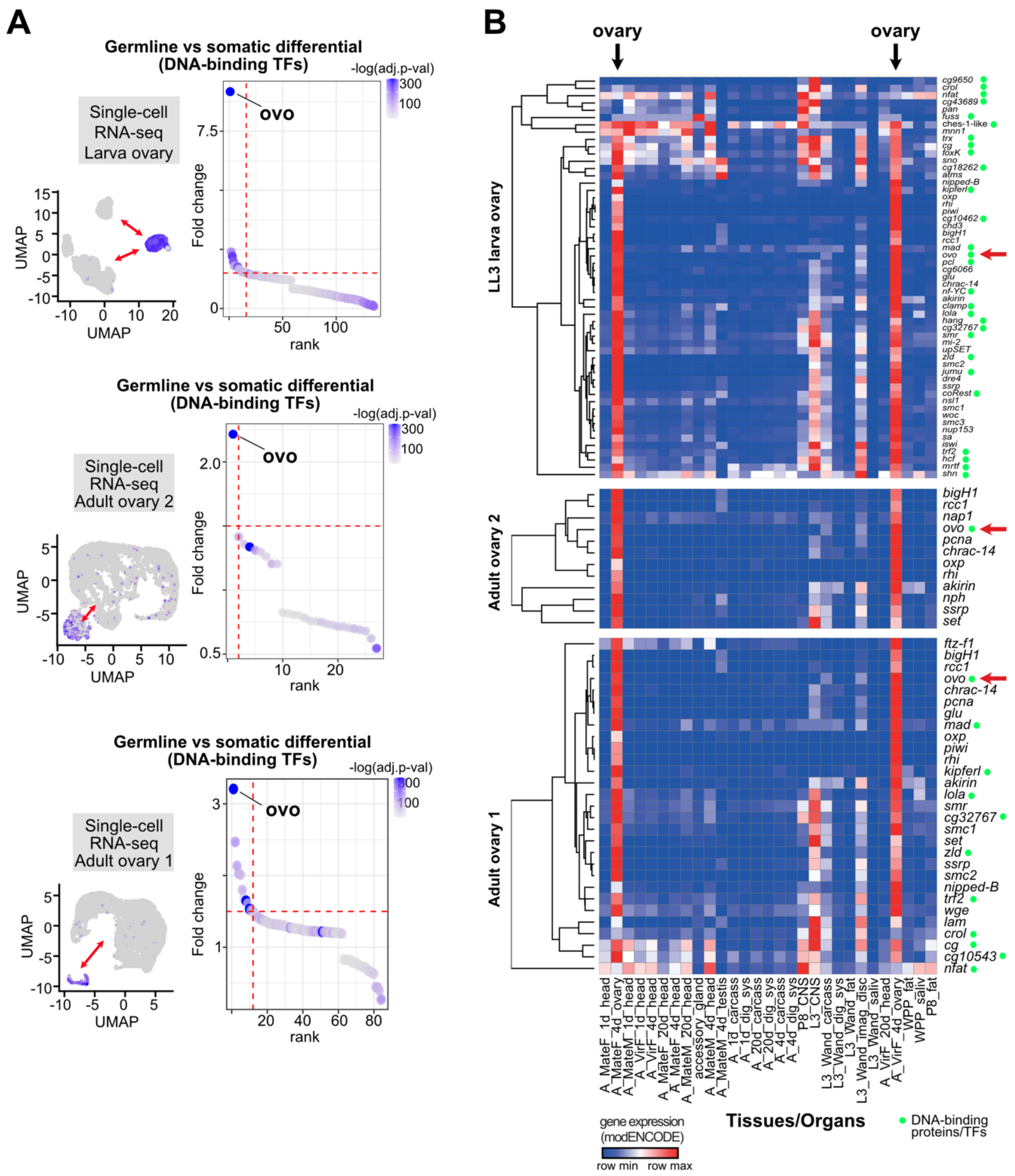

Supplemental Figure S3. Ovo is the major ovarian germline transcription factor in the *Drosophila* (also see Supplemental Dataset 2).
(A) Differential gene expression (Seurat package, FindMarkers tool) between the germline and somatic clusters of the LL3 larva and adult ovary single-cell RNA-seq datasets from (Jevitt et al. 2020; Rust et al. 2020; Slaidina et al. 2020) showing the top-ranking germline-enriched DNA-binding TFs. The colour scales show the non-parametric Wilcoxon rank sum test p-values adjusted with Bonferroni correction for multiple testing. (B) Cross-tissue expression (bulk RNA-seq, modENCODE) of the top-ranking chromatin-binding and DNA-binding candidate genes showing the highest co-expression with the germline piRNA factors in ovary single-cell RNA-seq datasets. Red arrows highlight *ovo* expression in ovaries.
