# Supplementary material for: Transcriptional regulation of the piRNA pathway by Ovo in animal ovarian germ cells
Source: Genes Dev. 2025 Feb 1;39(3-4):221–41. doi: 10.1101/gad.352120.124 (PMC11789646; doi:10.1101/gad.352120.124)
Supplement: Supplement 7 [file Supplemental_Figure_S4.pdf]

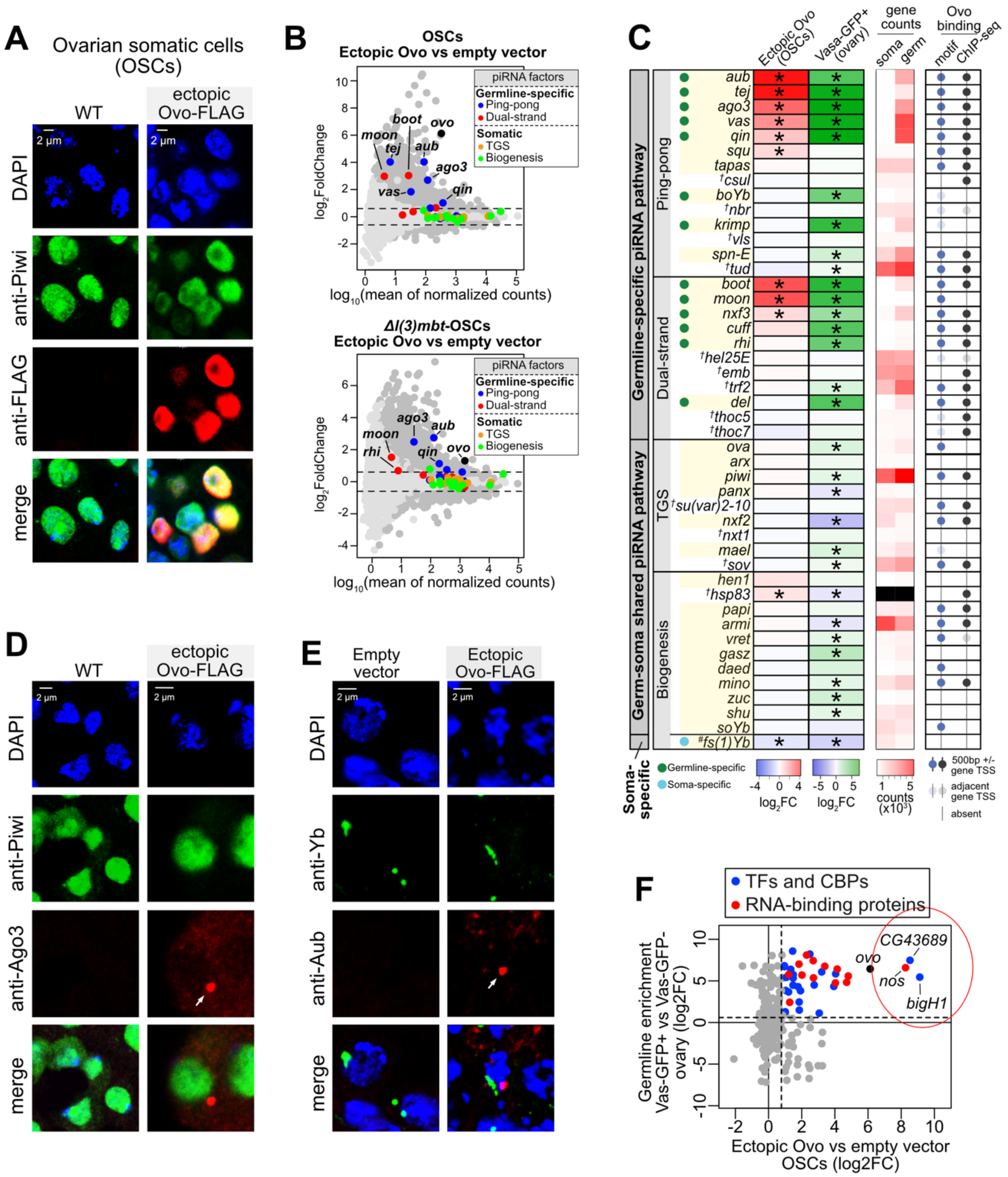

Supplemental Figure S4. Ectopic expression of *ovo-B* in ovarian somatic cells (OSCs) activates the expression of the germline-specific piRNA pathway components.

See next page for legend

Supplemental Figure S4. (continued from last page)
(A) Immunofluorescence images showing the nuclear localization of the Ovo-FLAG protein in OSCs after nucleofection with the *ovo-FLAG* construct (48-72 hr; Ovo-B isoform, NM_080338 transcript; blue=DAPI; green=Piwi; red=Ovo. (B) MA-plots showing the fold-changes and normalized counts of gene expression (DESeq2, RNA-seq n=3 replicates from distinct samples) for all genes following the nucleofection of the wild-type (WT) OSCs (top) and Δ*l(3)mbt* OSCs (bottom) with the *ovo-FLAG* construct (Ectopic Ovo) relative to the nucleofection with an empty vector. Data point colours: Light grey=not significant (DESeq2 adjusted p-value >0.1); Dark grey=significant (DESeq2 adjusted p-value <0.1). The piRNA pathway genes labelled according to the colour key (top right corners). TGS=transcriptional gene silencing. (C) Table summarising the fold-changes in gene expression for all the genes involved in the piRNA pathway (germline and somatic; TGS= transcriptional gene silencing) following the nucleofection with the *ovo-FLAG* construct) relative to the nucleofection with an empty vector in OSCs, and the fold-enrichments of the same genes in the *vas*-GFP+ germline cells compared to the *vas*-GFP- somatic cells from the FACS-sorted transgenic *vas*-GFP ovaries (DESeq2, RNA-seq n=3 replicates from distinct samples). The piRNA pathway-specific genes are highlighted in yellow; †=ubiquitous genes that are not specific to the piRNA pathway; #=somatic-specific piRNA pathway genes; *<0.01, adjusted p-values from DESeq2; normalized gene counts (DESeq2) are shown for *vas*-GFP- (soma) and *vas*-GFP+ (germ) cells (*hsp83* in black is excluded due to high expression levels). The presence of Ovo motifs and Ovo ChIP-seq peaks (ENCODE) within ±500 bp of gene TSS is shown with dots on the right side of the table. Transparent dots represent non-specific motifs and peaks within ±500 bp of gene TSS that are closer to a different/neighbouring gene. (D) Immunofluorescence images showing the presence of Ago3 proteins as peri-nuclear nuage-like foci (arrowheads) in the OSCs nucleofected with the ovo-FLAG construct (blue=DAPI; green=Piwi; red=Ago3). (E) Immunofluorescence images showing distinct localization of the nuage-like foci (red, Aub) appearing within the *ovo-FLAG* nucleofected OSCs from the somatic Yb bodies (green) normally present in OSCs. (F) Plot showing the Ovo target genes (x-axis; $\log_2$ fold-change response to ectopic Ovo expression in OSCs) that are germline-enriched (y-axis; *vas*-GFP+) and have gene-regulatory functions (TF=transcription factor, CBP=chromatin binding protein).
