# Supplementary material for: Transcriptional regulation of the piRNA pathway by Ovo in animal ovarian germ cells
Source: Genes Dev. 2025 Feb 1;39(3-4):221–41. doi: 10.1101/gad.352120.124 (PMC11789646; doi:10.1101/gad.352120.124)
Supplement: Supplement 8 [file Supplemental_Figure_S5.pdf]

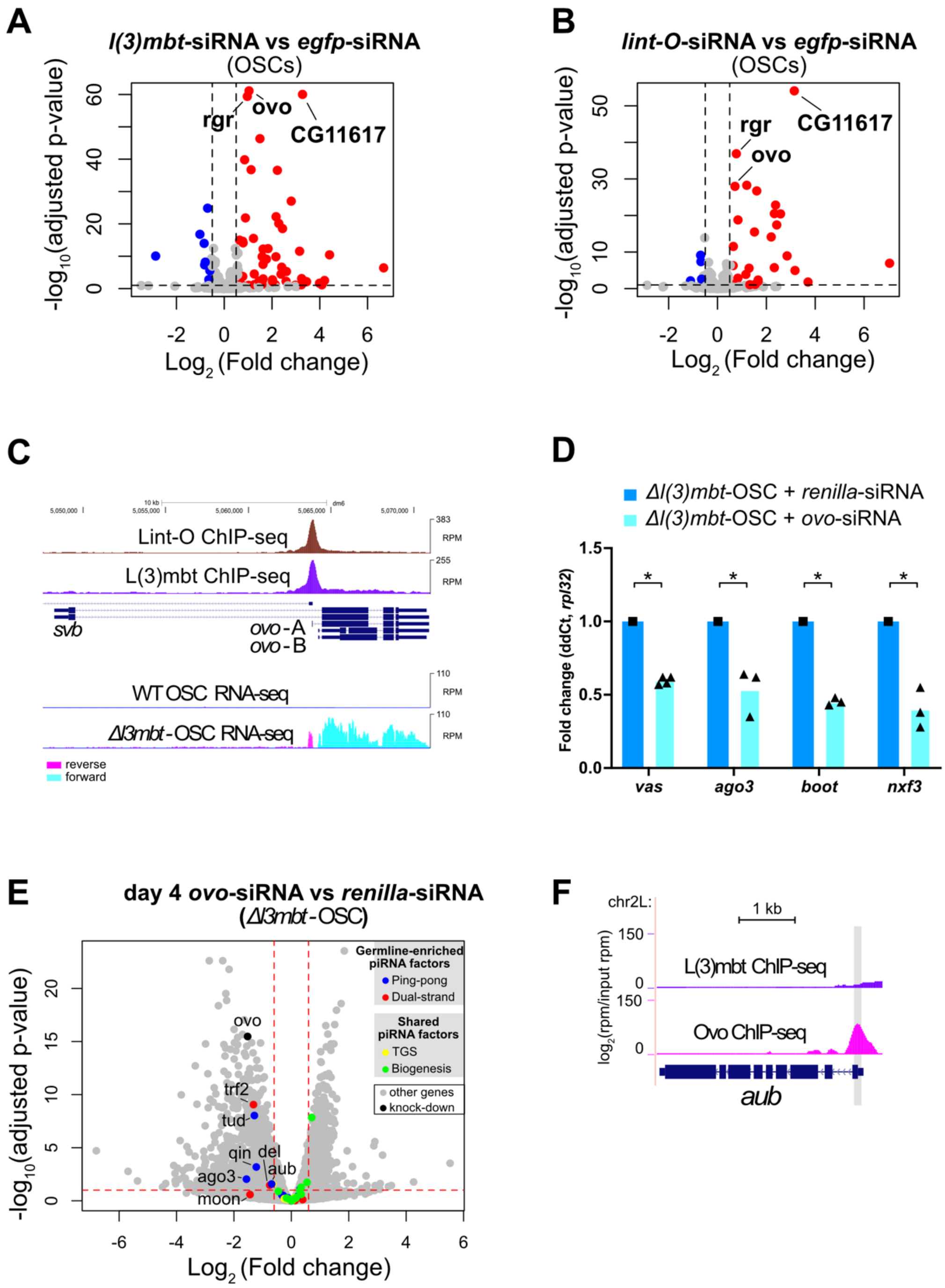

Supplemental Figure S5. The complex of L(3)mbt with Lint-O indirectly represses the expression of the germline piRNA pathway genes in ovarian somatic cells (OSCs) via inhibition of germline *ovo-B* transcription.
(A) siRNA knockdowns of *l(3)mbt* and (B) *lint-O* in ovarian somatic cells (OSCs) result in significant upregulation of *ovo*. (DESeq2; RNA-seq; n=3 replicates from distinct samples; data from (Yamamoto-Matsuda et al. 2022)). (C) Lint-O ChIP-seq showing a strong binding event at the germline *ovo* promoter (Ovo-B isoform) in OSCs where L(3)mbt ChIP-seq also shows a strong binding signal (rpm; merged n=2 replicates from distinct samples, data from (Yamamoto-Matsuda et al. 2022)). (D) *ovo* siRNA knockdown experiments in *Δl(3)mbt* OSCs (RT-qPCR; n≥3 replicates from distinct samples; p-value: *<0.01, one-tailed two-sample t-test). (E) Volcano plot showing downregulation of the germline-specific piRNA pathway genes on day 4 of *ovo* siRNA knock-downs in *Δl(3)mbt* OSCs using differential RNA-seq analysis (DESeq2) between *ovo* siRNA and *renilla* siRNA knockdowns (n=3 replicates from distinct samples). (F) L(3)mbt ChIP-seq from OSCs showing absence of L(3)mbt binding at the germline *aub* promoter (n=2 replicates from distinct samples; merged; data from (Yamamoto-Matsuda et al. 2022)). Ovo ChIP-seq showing Ovo binding to the *aub* promoter (n=2 replicates from distinct samples; merged; data from ENCODE, whole fly).
