# Supplementary material for: Transcriptional regulation of the piRNA pathway by Ovo in animal ovarian germ cells
Source: Genes Dev. 2025 Feb 1;39(3-4):221–41. doi: 10.1101/gad.352120.124 (PMC11789646; doi:10.1101/gad.352120.124)
Supplement: Supplement 9 [file Supplemental_Figure_S6.pdf]

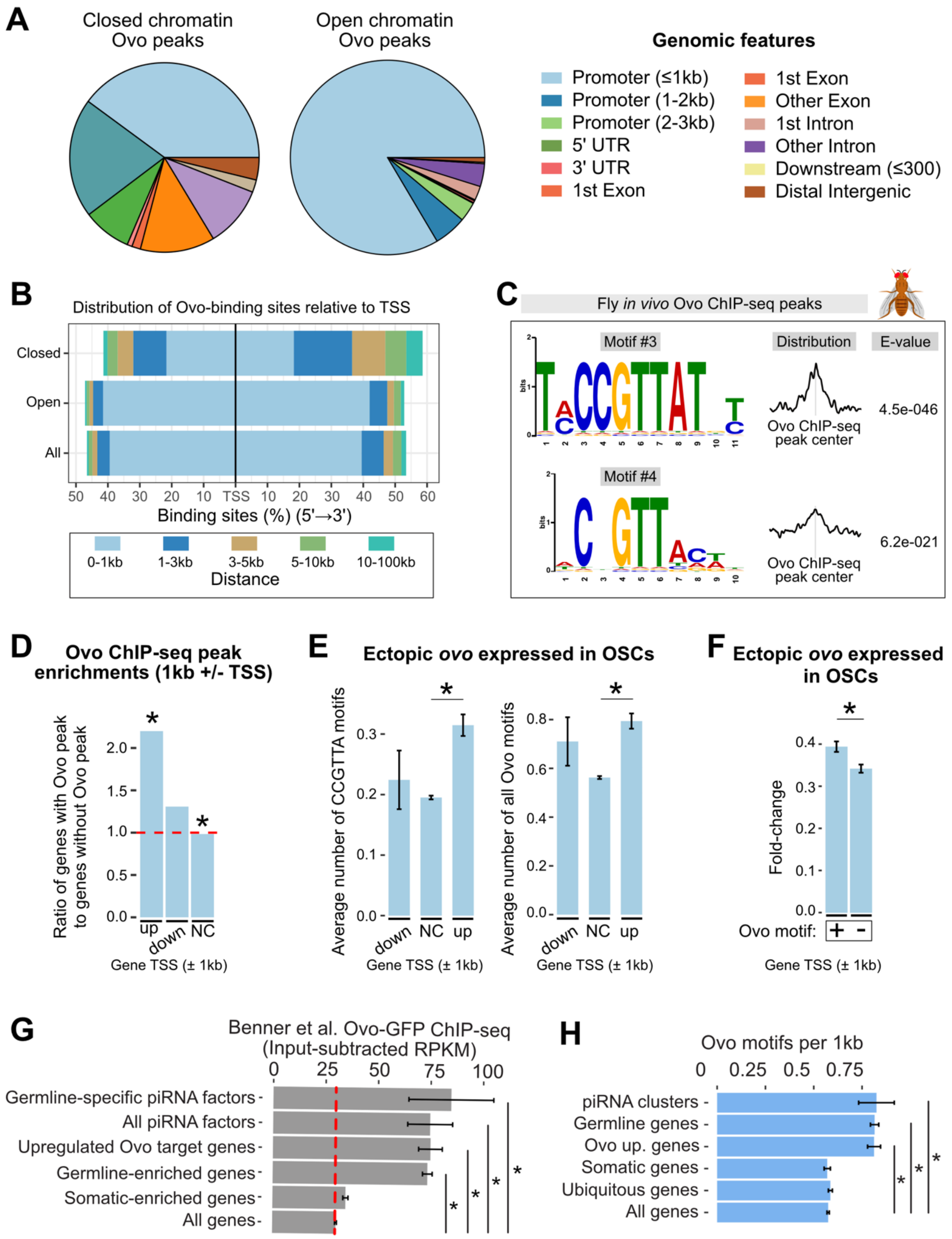

Supplemental Figure S6. Genomic features of Ovo binding sites in *Drosophila*.
See next page for legend

Supplemental Figure S6. (continued from last page)
(A) Genomic annotations (UCSC; Ensembl genes; dm6) of Ovo ChIP-seq peaks (data from ENCODE, whole fly) corresponding to open and closed chromatin states in fly ovaries based on ovary ATAC-seq peaks using ChIPseeker. (B) Distribution of open (ATAC-seq positive) and closed (ATAC-seq negative) Ovo ChIP-seq peaks relative to the gene transcription start sites (TSS). (C) The 3rd and 4th top-scoring *de novo* motifs discovered within fly Ovo ChIP-seq peaks using MEME-ChIP. (D) Enrichment of Ovo ChIP-seq peaks within ±1 kb of TSS of the genes that are upregulated, downregulated or do not change in expression in response to the ectopic Ovo expression in OSCs (p-value: *<0.01, Fisher's exact test). (E) Average number of CCGTTA Ovo motifs (B1H-derived; from FlyFactorSurvey database) and the average number of all Ovo motifs (both CCGTTA and CNGTTA motifs) within ±1 kb of TSS of genes that were downregulated (down), unresponsive (NC=no change), and upregulated (up) in response to the ectopic Ovo expression in OSCs. (F) Average fold-changes in response to the ectopic Ovo expression in OSCs compared between the genes that harbour (+) and lack (-) Ovo motif within ±1 kb of their TSS. Error bars indicate standard error of the mean. p-value: *<0.01, one-tailed two-sample t-test. (G) Ovary Ovo-GFP ChIP-seq signals at promoter regions of the piRNA pathway genes compared to upregulated Ovo target genes, germline-enriched genes, somatic-enriched genes and all genes (n=3, ChIP-seq data from (Benner et al. 2024)). (H) Numbers of Ovo motifs per 1 kb compared between piRNA clusters (±1 kb of ends) and promoter regions (±1 kb of TSS) of the germline-enriched genes, upregulated Ovo target genes, somatic-enriched genes, ubiquitously-expressed genes and all genes in *D.mel*.
