# Supplementary material for: Transcriptional regulation of the piRNA pathway by Ovo in animal ovarian germ cells
Source: Genes Dev. 2025 Feb 1;39(3-4):221–41. doi: 10.1101/gad.352120.124 (PMC11789646; doi:10.1101/gad.352120.124)
Supplement: Supplement 10 [file Supplemental_Figure_S7.pdf]

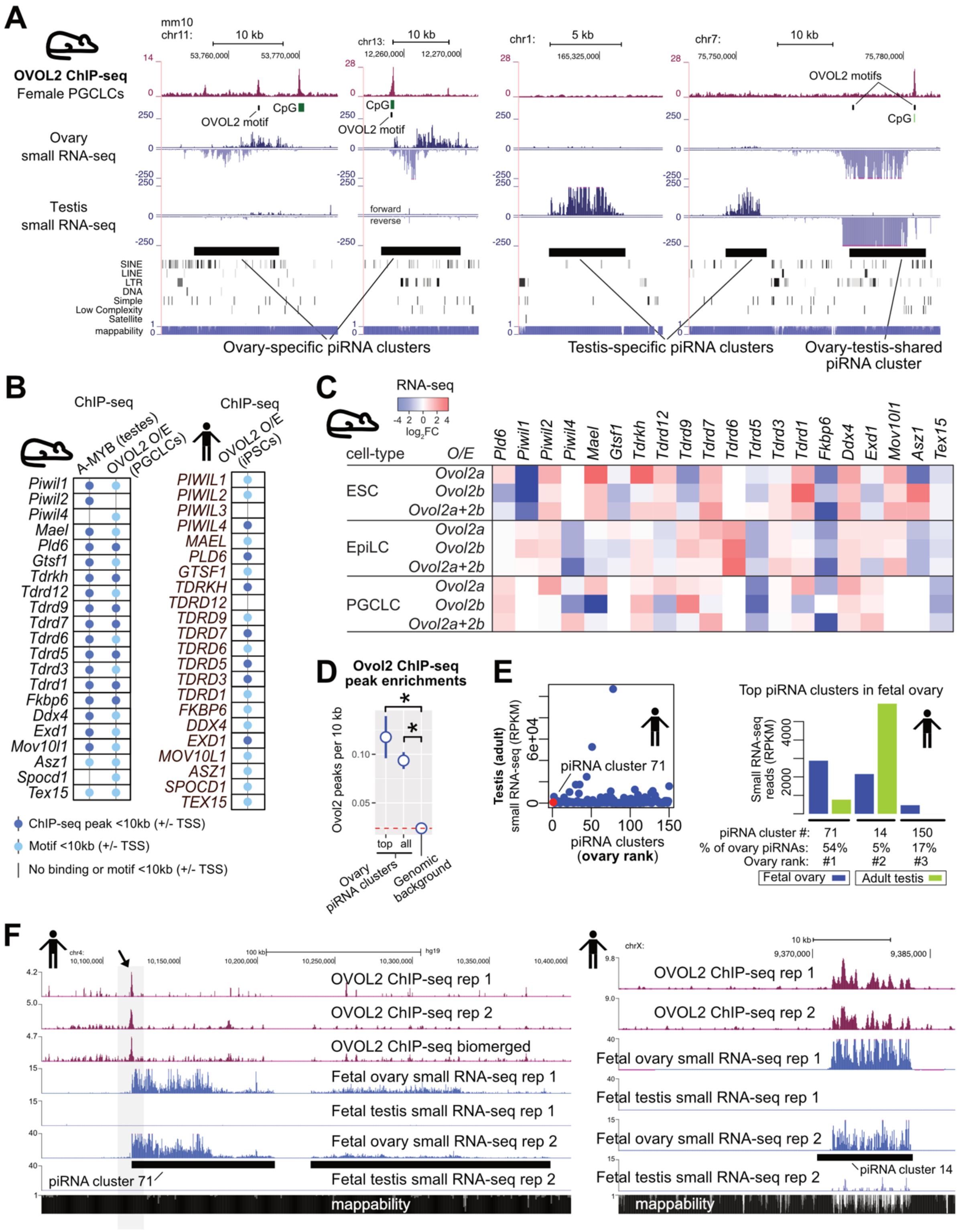

Supplemental Figure S7. Human and mouse OVOL2 binding to ovarian piRNA clusters.
See next page for legend

Supplemental Figure S7. (continued from last page)
(A) OVOL2 ChIP-seq (rpm; isoform 2A; merged n=2 replicates from distinct samples) from female mouse primordial germ cell-like cells (PGCLCs; day 2 of PGCLC induction; overexpressing transgenic mouse *Ovol2a*; data from (Naitou et al. 2022)) showing OVOL2 binding events at the mouse ovary piRNA clusters (small RNA-seq data from (Aravin et al. 2008)). ChIP-seq data for OVOL2A isoform is shown. The OVOL2B isoform shows the exact same binding patterns as OVOL2A. (B) Table summarizing binding of mouse A-MYB to piRNA factors in mouse testes (ChIP-seq data n=1 from (Li et al. 2013)), binding of mouse OVOL2 to piRNA factors in female mouse PGCLCs overexpressing (O/E) transgenic mouse *Ovol2a* (day 2 of PGCLC induction, OVOL2A isoform ChIP-seq data from (Naitou et al. 2022); merged n=2 replicates from distinct samples), and binding of human OVOL2 to piRNA factors in human induced pluripotent cell (iPSC) line WTC11 (male) overexpressing (O/E) human *OVOL2* (ChIP-seq data from ENCODE; merged n=2 replicates from distinct samples). (C) Changes in expression of mouse piRNA pathway genes (log_2 fold-change) under transgenic expression of mouse *Ovol2a* and *Ovol2b* throughout the *in vitro* differentiation of mouse embryonic stem cells (ESCs) to PGCLCs (day 2 of PGCLC induction) via epiblast-like cells (EpiLCs) (n=2 replicates from distinct samples, RNA-seq data from (Naitou et al. 2022)). (D) Numbers of mouse OVOL2 ChIP-seq peaks (isoform 2A, merged n=2 replicates from distinct samples; data from (Naitou et al. 2022)) per 10 kb at the piRNA clusters compared to the genomic background (all genomic regions that are not piRNA clusters). top=top-expressed ovary piRNA clusters (>20 rpm), all=all ovary piRNA clusters (>1 rpm). Error bars indicate standard error of the mean. p-value: *<0.05, Wilcoxon Signed-Rank Test. (E) The expression levels of the human piRNA clusters in the human adult testes ranked by their expression levels in human fetal ovaries (rpkm; data from (Williams et al. 2015)). Barplots (right) are showing the expression levels of the top 3 highest expressed human fetal ovary piRNA clusters (#71, #14 and #150) in fetal ovaries and adult testes (rpkm). % of ovary piRNAs indicates the percentage of all fetal ovary piRNA reads mapping to the indicated clusters (data from (Williams et al. 2015)). (F) OVOL2 ChIP-seq from human induced pluripotent cell (iPSC) line WTC11 overexpressing (O/E) ectopic OVOL2 construct (data from ENCODE) showing OVOL2 binding to the top expressed human fetal ovary piRNA clusters #71 (left) and #14 (right). Fetal ovary small-RNA-seq and fetal testes small-RNA-seq tracks are shown below (data from (Williams et al. 2015)). Mappability tracks from Duke Uniq 35 are shown below.
